# ADAPT-Net: an adaptive dynamic attention and persistence-aware transformer for overlapping community detection in complex social networks

**DOI:** 10.3389/frai.2026.1914215

**Published:** 2026-08-26

**Authors:** Gowthami Vusirikkayala, V. Madhu Viswanatham

**Affiliations:** School of Computer Science and Engineering, Vellore Institute of Technology, Vellore, Tamil Nadu, India

**Keywords:** complex social networks, dynamic graph learning, graph neural networks, overlapping community detection, persistence-aware transformer, temporal community detection

## Abstract

Overlapping community detection in dynamic complex networks is challenging because memberships evolve over time, interactions arrive irregularly, and genuine bridge roles must be distinguished from noisy multi-community assignments. The aim of this study is to develop and evaluate adaptive dynamic attention and persistence-aware transformer (ADAPT-Net), an adaptive dynamic attention and persistence-aware transformer for overlapping community detection in complex social networks. The proposed framework integrates adaptive event-driven graph partitioning, multiscale graph attention encoding, learnable node-centric scale fusion, cross-snapshot link-aware temporal attention, and density-preserving overlap regularization. The primary controlled evaluation uses one processed benchmark instance derived from the Real-world Dynamic Networks (DynaMo) collection. Under a unified five-fold held-out protocol, ADAPT-Net records an F1-score of 96.70 ± 0.65, precision of 95.75 ± 1.50, recall of 97.69 ± 0.49, Jaccard similarity of 93.62 ± 1.22, Omega Index of 88.91 ± 3.01, normalized mutual information (NMI) of 84.43 ± 2.59, area under the receiver operating characteristic curve (AUC–ROC) of 99.77 ± 0.11, and area under the precision–recall curve (AUPRC) of 99.59 ± 0.17. Additional label-independent structural validation is reported for Reddit, dblp computer science bibliography (DBLP), and Enron using modularity, coverage, conductance, internal density, overlap rate, mean memberships per node, and temporal switch rate. The controlled and external analyses address different questions: the processed DynaMo-derived benchmark instance measures agreement with the reference memberships contained in the evaluation folds, whereas the external networks assess structural cohesion and temporal stability without equivalent reference overlaps. The current implementation is trained offline on snapshot sequences and supports snapshot-level inference rather than fully online parameter updates.

## 1 Introduction

Community detection is a central problem in complex network analysis because it reveals groups of nodes that interact more strongly with one another than with the rest of the graph. In social, collaboration, communication, and information-sharing networks, such groups help explain interaction patterns, influence routes, collective behavior, and role formation (Nooribakhsh et al., 2024; Gmati et al., 2024; Huang et al., 2024; Zhang et al., 2023). However, real social systems are rarely static and strictly disjoint. A user, organization, or entity can participate in several groups at the same time; therefore, overlapping community detection is needed to represent bridge nodes, shared memberships, and multi-role participation (Ji et al., 2024, 2023; Cai et al., 2024).

Dynamic networks make this problem more difficult. Edges may appear, disappear, intensify, or weaken across time, and community boundaries may shift as new interactions emerge. A common practice is to convert a temporal edge stream into snapshots using fixed time windows. This strategy is simple, but it can split one meaningful interaction burst into several graphs or merge unrelated interactions into the same snapshot. Such temporal mismatch can weaken the quality of community assignments, especially when interactions are irregular or burst-driven (Costa and Ralha, 2023).

Existing overlapping community detection approaches include optimization-based methods, metaheuristic algorithms, fuzzy clustering, graph convolutional models, autoencoder-based models, graph autoencoders, label-propagation mechanisms, and common-neighbor-based methods (Heydariyan et al., 2024; Gharehchopogh, 2023; Al-Andoli et al., 2024; Yuan et al., 2023; Khawaja et al., 2025; Kumar et al., 2023; Joshi and Singh, 2025; Tang et al., 2025). These approaches provide useful foundations for community assignment and overlap modeling, but many of them are designed for static graphs or independently processed snapshots. They do not fully model persistent temporal relationships, and they may produce overlapping assignments without checking whether the predicted overlaps are supported by sufficient graph connectivity.

The primary controlled experiments use one processed benchmark instance derived from the Real-world Dynamic Networks (DynaMo) collection (Zhuang et al., 2021). The instance is evaluated using a common snapshot-construction, chronological splitting, training, and held-out evaluation protocol. Graph representation learning has improved the ability to model complex network structure. Node2Vec captures structural proximity through biased random walks, while the Graph Convolutional Network (GCN) and Graph Attention Network (GAT) learn node representations through message passing and neighborhood aggregation (Grover and Leskovec, 2016; Kipf and Welling, 2017; Veličković et al., 2018). Graph Attention Network version 2 (GATv2) further improves graph attention by making the attention ranking more expressive (Brody et al., 2022). Hierarchical pooling methods such as Differentiable Pooling (DiffPool) provide a way to capture coarse-grained graph structure (Ying et al., 2018). Transformer attention and time-aware encoding further motivate temporal representation learning for evolving graphs (Vaswani et al., 2017; Kazemi et al., 2019).

Cluster affiliation model for big networks (BigCLAM) is a scalable affiliation-factor model for overlapping communities, while neural overlapping community detection (NOCD) extends this direction using graph neural networks (Yang and Leskovec, 2013; Shchur and Günnemann, 2019). For evolving graphs, DynAERNN, dynamic self-attention network (DySAT), evolving graph convolutional network (EvolveGCN), and DyGFormer introduce different forms of temporal graph learning (Goyal et al., 2020; Sankar et al., 2020; Pareja et al., 2020; Yu et al., 2023). These methods provide important baselines, but dynamic overlapping community detection still requires explicit handling of temporal irregularity, heterogeneous node roles, persistent interactions, and structurally meaningful overlap formation.

To address these issues, this paper proposes ADAPT-Net, an adaptive dynamic attention and persistence-aware transformer framework for overlapping community detection in complex social networks. The framework combines adaptive event-driven graph partitioning, multiscale graph attention encoding, learnable node-centric scale fusion, cross-snapshot link-aware temporal attention, and density-preserving overlap regularization. The event-driven partitioning module constructs snapshots from changes in edge-arrival behavior rather than relying only on fixed windows. The multiscale encoder captures both local neighborhood structure and coarse community-level context. The node-centric fusion module learns how much local or global information each node requires. The temporal transformer incorporates link persistence, and the overlap regularization term discourages weakly supported multi-community assignments.

A controlled benchmark is useful for consistent fold-wise comparison, but one benchmark alone cannot establish broad generalization. The study therefore includes Reddit, DBLP, and Enron as external dynamic networks spanning social interaction, scientific collaboration, and email communication. These networks do not provide equivalent fold-wise overlapping reference memberships to those contained in the processed DynaMo-derived evaluation folds. Their label-independent structural and temporal measures are therefore interpreted separately from the processed DynaMo-derived benchmark agreement metrics and are not treated as interchangeable evidence. The main contributions of this work are summarized as follows:

- An adaptive event-driven graph partitioning strategy is introduced to construct temporal snapshots from irregular interaction streams without depending only on fixed-window slicing.
- A multiscale graph attention encoder is used to learn local neighborhood representations and coarse-grained structural representations for dynamic social networks.
- A learnable node-centric scale fusion mechanism is designed to combine fine-grained and coarse-grained representations according to node-specific structural roles.
- A cross-snapshot link-aware temporal transformer is developed to incorporate relational persistence into temporal attention for dynamic overlapping community modeling.
- A density-preserving overlap regularization term is introduced to reduce multi-community assignments that lack sufficient structural support.
- A unified held-out test evaluation is conducted against GCN, GAT, BigCLAM, NOCD, DynAERNN, DySAT, EvolveGCN, and DyGFormer using consistent metric definitions and fold-wise reporting.

The remainder of this paper is organized as follows. Section 2 reviews community detection, overlapping community detection, graph representation learning, and dynamic graph neural networks. Section 3 describes the proposed ADAPT-Net framework. Section 4 presents the experimental results and discussion. Section 5 discusses the study limitations, and Section 6 concludes the paper and outlines future research directions.

## 2 Related work

### 2.1 Community detection in complex networks

Community detection is widely used to identify densely connected groups in complex networks. In social networks, detected communities can reveal interaction groups, shared interests, collaboration patterns, influence paths, and coordinated behavior. Traditional methods often rely on graph partitioning, modularity optimization, label propagation, similarity measures, or matrix factorization. Recent work has increasingly used machine learning and deep learning to improve representation quality, scalability, and robustness in large social networks (Nooribakhsh et al., 2024; Gmati et al., 2024; Huang et al., 2024; Zhang et al., 2023).

Recent studies have also examined influence maximization, graph analytics, nonnegative matrix factorization, semi-supervised learning, and structure obfuscation in community detection and complex network analysis (Kazemzadeh et al., 2023; Ali et al., 2023; Liu et al., 2024; Berahmand et al., 2024; Zhao and Cheong, 2023). These studies show that community detection remains an active problem across social, information, and interaction networks, but most of them do not jointly address dynamic evolution, overlapping membership, and temporal persistence.

Disjoint community detection remains useful, but it is limited when nodes naturally participate in several groups. In many social systems, a node may simultaneously belong to professional, academic, social, and interest-based communities. Overlapping community detection is therefore important for modeling shared roles, bridge nodes, information diffusion, and multi-group participation (Ji et al., 2024, 2023; Cai et al., 2024). The challenge is that the model must identify both community boundaries and shared membership regions.

### 2.2 Overlapping community detection

Overlapping community detection has been studied using optimization, fuzzy clustering, matrix factorization, and deep learning. Evolutionary and metaheuristic methods optimize community-quality functions and can detect overlapping structures in complex networks (Heydariyan et al., 2024; Gharehchopogh, 2023). Graph convolutional and fuzzy-clustering models combine representation learning with soft assignment to improve overlapping membership prediction (Al-Andoli et al., 2024; Yuan et al., 2023). Other approaches use autoencoders, graph autoencoders, or label-propagation mechanisms to learn latent graph structure before assigning communities (Kumar et al., 2023; Joshi and Singh, 2025; Tang et al., 2025).

Common-neighbor-based approaches have also been studied for overlapping community detection, but they mainly depend on structural proximity and do not directly model temporal membership evolution (Khawaja et al., 2025).

BigCLAM is a widely used scalable baseline for overlapping community detection. It represents each node through nonnegative affiliation strengths and estimates edge likelihood from shared community memberships (Yang and Leskovec, 2013). NOCD extends overlapping community detection using graph neural networks and community affiliation learning (Shchur and Günnemann, 2019). Although these models are useful for overlap modeling, they are mainly designed for static graphs or snapshot-level processing. In dynamic networks, this may limit their ability to capture persistent relationships and evolving membership patterns.

### 2.3 Graph representation learning for community detection

Graph representation learning maps nodes into low-dimensional vectors while preserving relational information. Node2Vec learns structural features using biased random walks and captures both neighborhood proximity and broader structural roles (Grover and Leskovec, 2016). Graph convolutional networks learn node embeddings by aggregating information from local neighborhoods (Kipf and Welling, 2017). Graph attention networks improve this process by assigning adaptive weights to neighboring nodes during message passing (Veličković et al., 2018). GATv2 further improves attention expressiveness by addressing limitations of static attention ranking (Brody et al., 2022).

Community detection often benefits from both local and global structural information. Local aggregation captures immediate neighborhood patterns, whereas coarse-grained representations provide broader community-level context. Hierarchical pooling methods such as DiffPool learn soft assignments from nodes to latent clusters and can capture coarse graph organization (Ying et al., 2018). However, a fixed balance between local and global structure may not suit all nodes. Hub nodes, bridge nodes, and peripheral nodes can require different structural scales, which motivates node-centric fusion.

### 2.4 Dynamic graph neural networks

Dynamic graph neural networks extend graph learning to networks whose edges, nodes, or attributes change over time. DynAERNN combines structural encoding with recurrent temporal learning to capture changes in graph structure (Goyal et al., 2020). DySAT models dynamic graphs using structural and temporal self-attention across snapshots (Sankar et al., 2020). EvolveGCN updates graph convolutional parameters over time so that the model evolves with the graph (Pareja et al., 2020). DyGFormer uses a transformer-based design for dynamic graph representation learning and provides a recent temporal graph baseline (Yu et al., 2023).

These models show that temporal learning can improve representation quality in evolving networks. However, many dynamic graph methods are designed primarily for link prediction, node classification, or general representation learning. They do not always explicitly target overlapping community assignments. In addition, snapshot construction is often based on fixed intervals, which may not match burst-driven social interaction patterns. Dynamic overlapping community detection therefore requires both adaptive temporal graph construction and overlap-aware representation learning.

### 2.5 Research gap and motivation

The comparison in Table 1 shows that existing methods address different parts of the dynamic overlapping community detection problem. BigCLAM and NOCD directly model overlapping affiliations, but they do not explicitly address event-driven temporal graph construction or persistence-aware temporal attention. GCN and GAT-based models are useful for graph representation learning, but their original formulations are not designed for dynamic overlapping community detection. Dynamic graph models such as DynAERNN, DySAT, EvolveGCN, and DyGFormer capture temporal evolution, but they generally do not enforce density-supported overlapping community assignments.

**Table 1:** Comparison of related methods with the proposed ADAPT-Net framework.

Method	Main category	Overlapping CD	Dynamic graph	Limitation addressed by ADAPT-Net
BigCLAM (Yang and Leskovec, 2013)	Overlapping community detection	Yes	No	Does not model temporal graph evolution or persistence-aware relationships.
NoCD (Shchur and Günnemann, 2019)	GNN-based overlapping community detection	Yes	No	Does not include adaptive temporal partitioning or link-persistence-aware attention.
GCN (Kipf and Welling, 2017)	Static graph neural network	No	No	Not designed for dynamic overlapping community assignment.
GAT (Veličković et al., 2018)	Attention-based graph neural network	No	No	Does not explicitly model temporal evolution or overlap-specific regularization.
DynAERNN (Goyal et al., 2020)	Dynamic graph representation learning	No	Yes	Does not explicitly optimize overlapping memberships.
DySAT (Sankar et al., 2020)	Dynamic graph attention	No	Yes	Does not include density-preserving overlap regularization.
EvolveGCN (Pareja et al., 2020)	Dynamic graph neural network	No	Yes	Does not directly enforce structurally supported overlaps.
DyGFormer (Yu et al., 2023)	Dynamic graph transformer	No	Yes	Primarily targets dynamic representation learning rather than overlap-valid assignments.
ADAPT-Net	Proposed framework	Yes	Yes	Combines event-driven partitioning, multiscale attention, node-centric fusion, persistence-aware temporal attention, and DPOR.

Three gaps remain important. First, fixed-window graph construction may fail to preserve burst-driven temporal structure. Second, uniform aggregation or fixed fusion may not represent heterogeneous node roles effectively. Third, overlapping memberships may be assigned without verifying whether the predicted overlap is structurally supported by sufficient connectivity. ADAPT-Net addresses these gaps by combining event-driven graph partitioning, multiscale graph attention, node-centric fusion, link-persistence-aware temporal attention, and density-preserving overlap regularization.

## 3 Methodology

**Figure 1:**
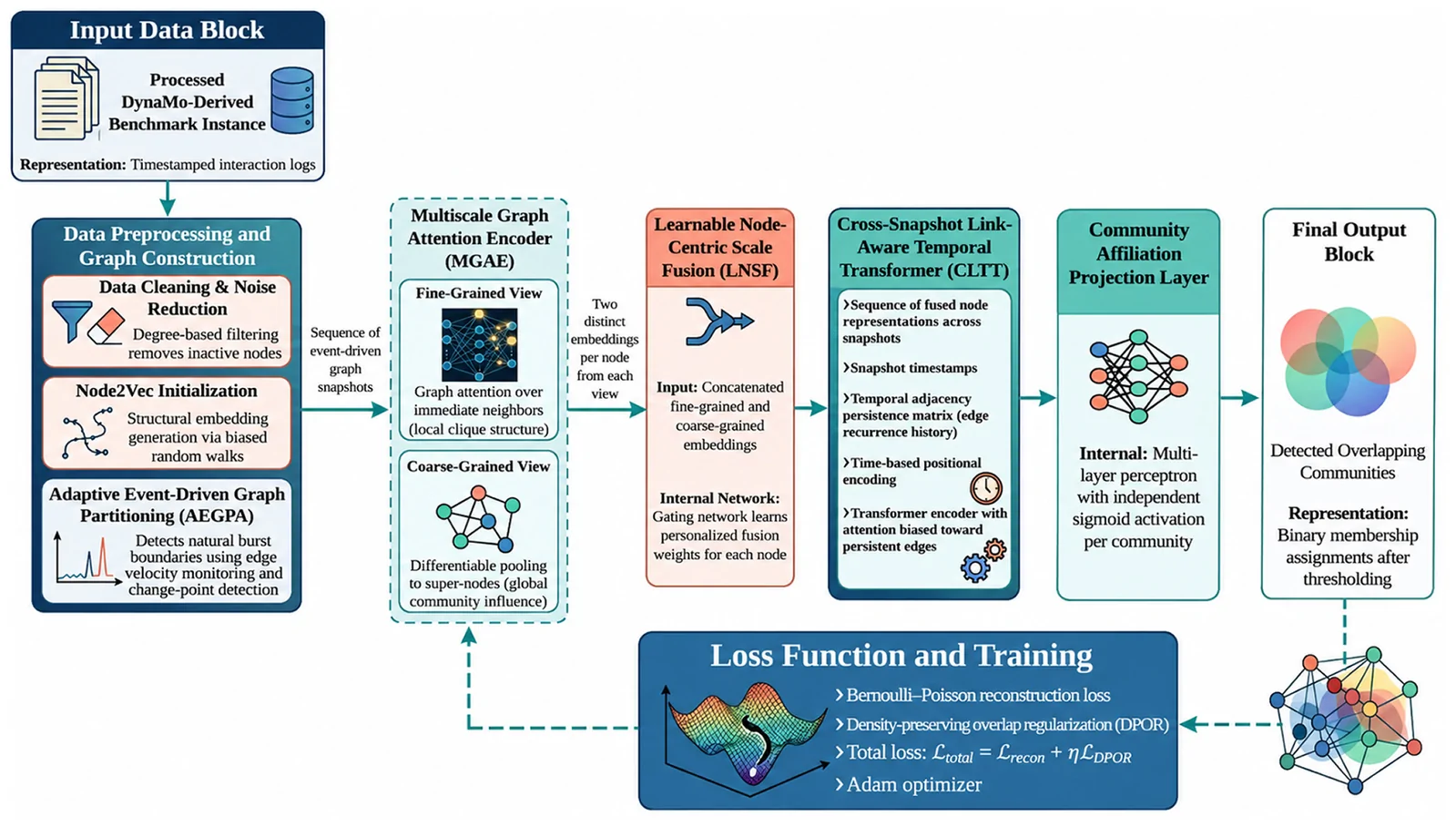
Overall workflow of the proposed ADAPT-Net framework. *L*_total_ = *L*_recon_+*ηL*_DPOR_ and the Adam optimizer.

**Figure 2:**
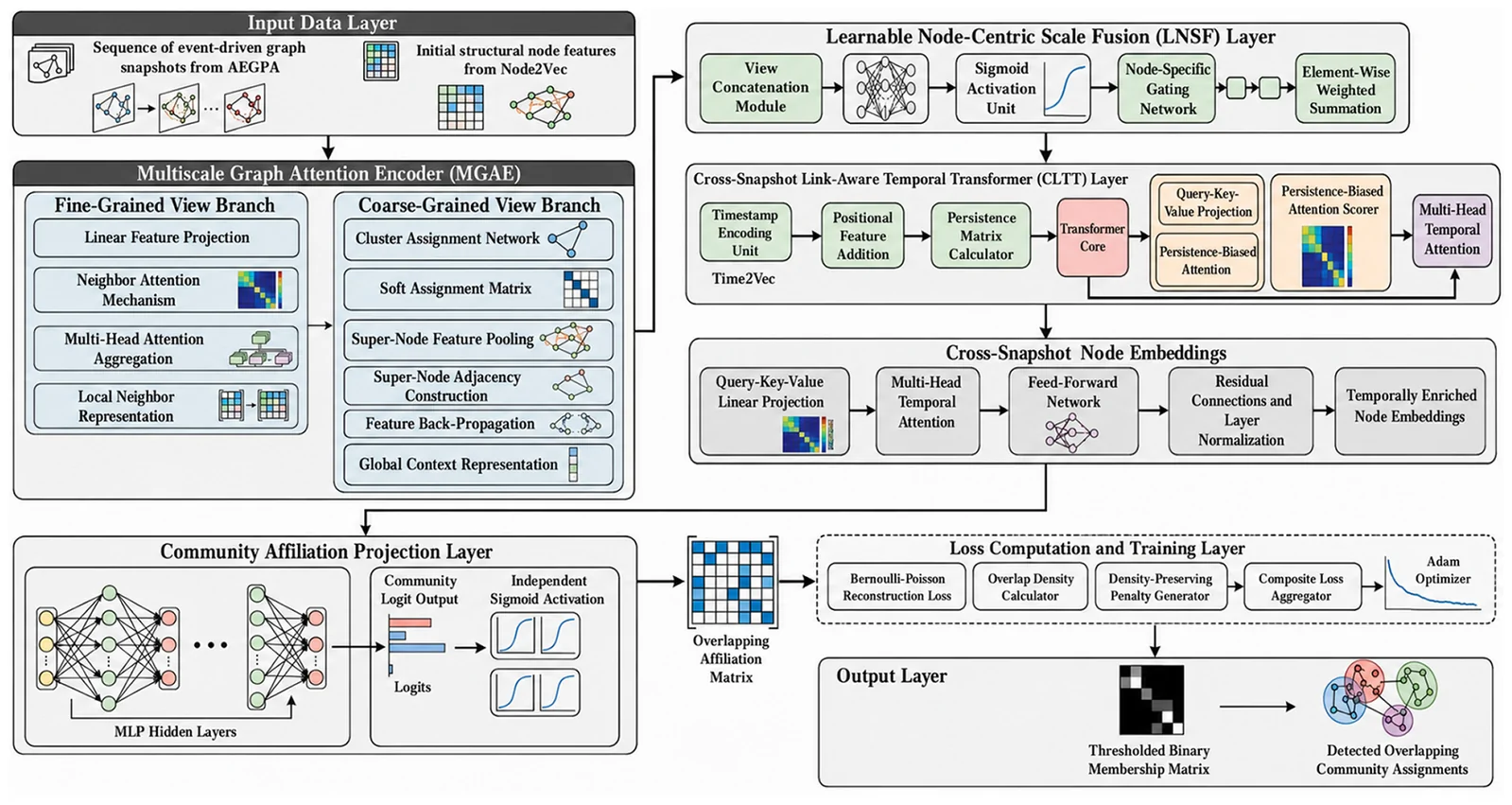
Detailed architecture of the proposed ADAPT-Net model.

This section presents the proposed ADAPT-Net framework for overlapping community detection in dynamic complex social networks. The methodology preserves the original ADAPT-Net design: input data acquisition, data preprocessing and graph construction, Adaptive Event-Driven Graph Partitioning Algorithm (AEGPA), Multiscale Graph Attention Encoder (MGAE), Learnable Node-Centric Scale Fusion (LNSF), Cross-Snapshot Link-Aware Temporal Transformer (CLTT), Community Affiliation Projection, Density-Preserving Overlap Regularization (DPOR), and the final training objective. The overall workflow is shown in Figure 1, and the detailed architecture is shown in Figure 2. Table 2 consolidates the notation used in the equations.

**Table 2:** Principal notation used in ADAPT-Net.

Symbol	Definition
*N, E, T*	Number of nodes, mean number of edges per snapshot, and number of snapshots.
*n*_int_	Number of recorded timestamped interactions.
*d, H, d*_*k*_	Hidden dimension, number of graph-attention heads, and query/key dimension.
*C, W, K*	DiffPool cluster count, CLTT temporal history window, and number of affiliation outputs.
*w*	AEGPA trailing window used to estimate recent edge-velocity variation.
$E_{\tau },E_{\tau }^{-}$	Observed edge set and sampled non-edge set for snapshot τ.
*M*^(τ)^∈[0, 1]^*N*×*K*^	Soft affiliation-probability matrix at snapshot τ.
*P* ^(τ)^	Link-persistence matrix; Bernoulli–Poisson edge probabilities are described separately in the reconstruction objective.
$S_{i}^{\text{CUSUM}}$	CUSUM change statistic at interaction index *i*.
*I*[·]	Indicator function, equal to one when the stated condition is true and zero otherwise.
θ, λ, η	Inference threshold, persistence strength, and DPOR loss weight.
δ_min_, ϵ	Minimum supported overlap density and numerical stabilizer.

The ADAPT-Net modules are integrated sequentially as follows. The timestamped interaction stream is first filtered and partitioned by AEGPA into event-driven graph snapshots *G*_τ_. Node2Vec provides the initial node-feature matrix $X_{\tau }^{\left(0\right)}$ for each snapshot. MGAE then processes these features through a fine-grained graph-attention branch and a coarse-grained DiffPool branch. LNSF combines the resulting local and global representations to obtain the fused snapshot embedding *Z*^(τ)^. CLTT subsequently incorporates temporal encoding and link persistence across the bounded history window to produce the temporally enriched representation $H_{\text{temp}}^{\left(\tau \right)}$. The community-affiliation projection layer maps this representation to the soft membership matrix *M*^(τ)^. During training, the Bernoulli–Poisson reconstruction loss and DPOR loss are jointly optimized. During inference, the membership threshold and fallback rule convert the soft affiliation probabilities into the final overlapping community assignments.

### 3.1 Input data acquisition

The primary controlled input is one processed benchmark instance derived from the Real-world Dynamic Networks (DynaMo) collection (Zhuang et al., 2021). The processed evaluation folds used in this study contain timestamped interaction records and associated reference membership matrices. Each fold contains 500 nodes, five reference affiliation dimensions, and 20 temporal snapshots. Among the 500 nodes, 125 nodes have more than one active reference membership, corresponding to an overlapping-node fraction of 0.25. These processed reference memberships are used only for evaluation and are not included in the ADAPT-Net training objective. The temporal edge sequence of the processed instance is represented as:


$$\begin{array}{l} E_{\text{cum}}={\left\{\left(u_{i},v_{i},t_{i}\right)\right\}}_{i=1}^{n_{\text{int}}}. \end{array}$$ (1)

Here, *u*_*i*_ and *v*_*i*_ are interacting nodes, *t*_*i*_ is the interaction timestamp, and *n*_int_ is the number of recorded interactions. The cumulative graph is


$$\begin{array}{l} G_{\text{cum}}=\left(V,E_{\text{cum}}\right), \end{array}$$ (2)

where *V* is the node set. Chronological causality is preserved using 60 percent training, 20 percent validation, and 20 percent held-out testing portions. The controlled protocol uses 500 nodes, five reference affiliation dimensions, *K* = 5 affiliation outputs, *C* = 20 internal DiffPool clusters, 20 AEGPA snapshots, and five held-out folds. Table 3 summarizes the benchmark configuration and evaluation protocol.

**Table 3:** Statistics and evaluation protocol of the processed DynaMo-derived benchmark instance.

Property	Value
Source collection	Real-world dynamic networks (DynaMo)
Processed benchmark instances evaluated	1
Nodes per processed fold	500
Reference affiliation dimensions in processed folds	5
Affiliation output dimension *K*	5
DiffPool internal cluster count *C*	20
Temporal split	60% training, 20% validation, and 20% held-out testing
Evaluation protocol	Five-fold held-out evaluation
Membership threshold θ	0.50
Random seeds	1, 2, 3, 4, and 5
Mean number of edges per snapshot	4, 608.5 ± 663.08
Edge-snapshot occurrences per fold	Approximately 92,170
Number of snapshots	20
Temporal span	20 normalized discrete temporal steps
Reference overlapping-node fraction in processed folds	0.25, corresponding to 125 of 500 nodes
Constructed graph type	Undirected and unweighted

4, 608.5 × 20 = 92, 170 is a rounded derived count of edge-snapshot occurrences, not a unique cumulative-edge count. The five reference affiliation dimensions and the 0.25 overlapping-node fraction describe the processed evaluation folds used in this study. They are not presented as general properties of the complete DynaMo collection.

For external structural validation, Reddit (Hamilton et al., 2017) is represented using monthly social-interaction networks, DBLP (Benson et al., 2018) is represented as a temporal co-authorship network in which publication years define the interaction timestamps, and Enron (Klimt and Yang, 2004) is represented using dated email interactions. For DBLP, publications were converted into pairwise co-authorship interactions and ordered by publication year. For Enron, sender–recipient interactions were extracted from the email records and ordered by message timestamp. Each temporal sequence was chronologically partitioned into snapshots before inference. Equivalent overlapping reference labels were not assumed for these networks; consequently, the external analysis used label-independent structural and temporal measures rather than processed-fold membership-agreement metrics.

### 3.2 Data preprocessing and graph construction

The preprocessing pipeline consists of degree-based filtering, structural feature initialization, and adaptive event-driven graph partitioning.

#### 3.2.1 Data cleaning and noise reduction

Low-activity nodes are removed because they may add noise with limited structural value. The cumulative degree of a node is computed as:


$$\begin{array}{l} \deg_{\text{cum}}\left(v\right)=|\left\{\left(u,w,t\right)\in E_{\text{cum}}|u=v\;\text{or }w=v\right\}|. \end{array}$$ (3)

The retained node set is defined as:


$$\begin{array}{l} V^{\prime }=\left\{v\in V|\deg_{\text{cum}}\left(v\right)\geq \theta_{\deg}\right\}. \end{array}$$ (4)

The filtered edge set is given by:


$$\begin{array}{l} E_{\text{cum}}^{\prime }=\left\{\left(u,v,t\right)\in E_{\text{cum}}|u\in V^{\prime },\;v\in V^{\prime }\right\}. \end{array}$$ (5)

The filtered cumulative graph is defined as:


$$\begin{array}{l} G_{\text{cum}}^{\prime }=\left(V^{\prime },E_{\text{cum}}^{\prime }\right). \end{array}$$ (6)

#### 3.2.2 Structural feature initialization using Node2Vec

Node2Vec is used to initialize node features on $G_{\text{cum}}^{\prime }$, because biased random walks capture neighborhood proximity and structural roles (Grover and Leskovec, 2016). For a walk moving from previous node *r* to current node *v* and candidate node *x*, the transition probability is defined as:


$$\begin{array}{l} \mathrm{Pr}\left(x|v,r\right)=\frac{\pi_{vx}}{\sum_{x^{\prime }\in N\left(v\right)}\pi_{vx^{\prime }}},\;\pi_{vx}=\alpha_{pq}\left(r,x\right)w_{vx}. \end{array}$$ (7)

The search-bias term is given by:


$$\begin{array}{l} \alpha_{pq}\left(r,x\right)=\{\begin{array}{ll} 1/p, & d_{rx}=0,\\ 1, & d_{rx}=1,\\ 1/q, & d_{rx}=2. \end{array} \end{array}$$ (8)

The Skip-gram negative sampling objective is given by:


$$\begin{array}{l} L_{\text{N2V}}=-\underset{u\in V^{\prime }}{\sum }\left[\underset{c\in N_{\text{walk}}\left(u\right)}{\sum }\log\sigma \left(h_{u}^{T}h_{c}\right)+\overset{K_{n}}{\underset{s=1}{\sum }}E_{n\sim P_{n}}\log\sigma \left(-h_{u}^{T}h_{n}\right)\right]. \end{array}$$ (9)

The resulting initial feature matrix is given by:


$$\begin{array}{l} X^{\left(0\right)}\in {\text{R}}^{|V^{\prime }|\times d}. \end{array}$$ (10)

#### 3.2.3 Adaptive event-driven graph partitioning

**Figure 3:**
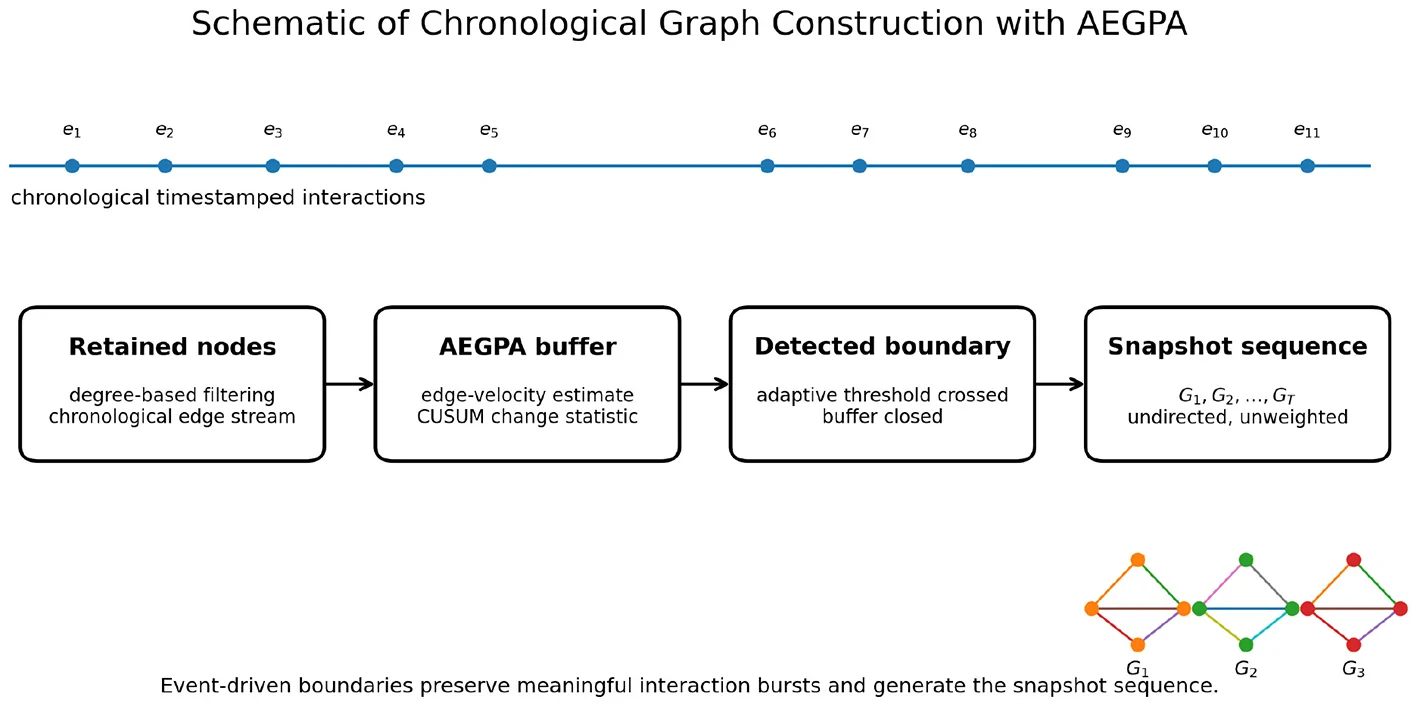
Visualization of the graph-construction process, showing chronological interactions, retained-node filtering, AEGPA buffering, adaptive change detection, snapshot-boundary identification, and the resulting dynamic graph snapshots.

AEGPA divides dynamic interaction streams by detecting statistically significant variations in edge-formation rates using a Cumulative Sum (CUSUM)-based change statistic instead of fixed time windows. The pseudocode is given in Algorithm 1. Figure 3 provides a schematic view of the chronological interaction stream, the AEGPA buffer, the adaptive change statistic, the detected boundary, and the resulting snapshot sequence.

Algorithm 1 Pseudocode of AEGPA. 
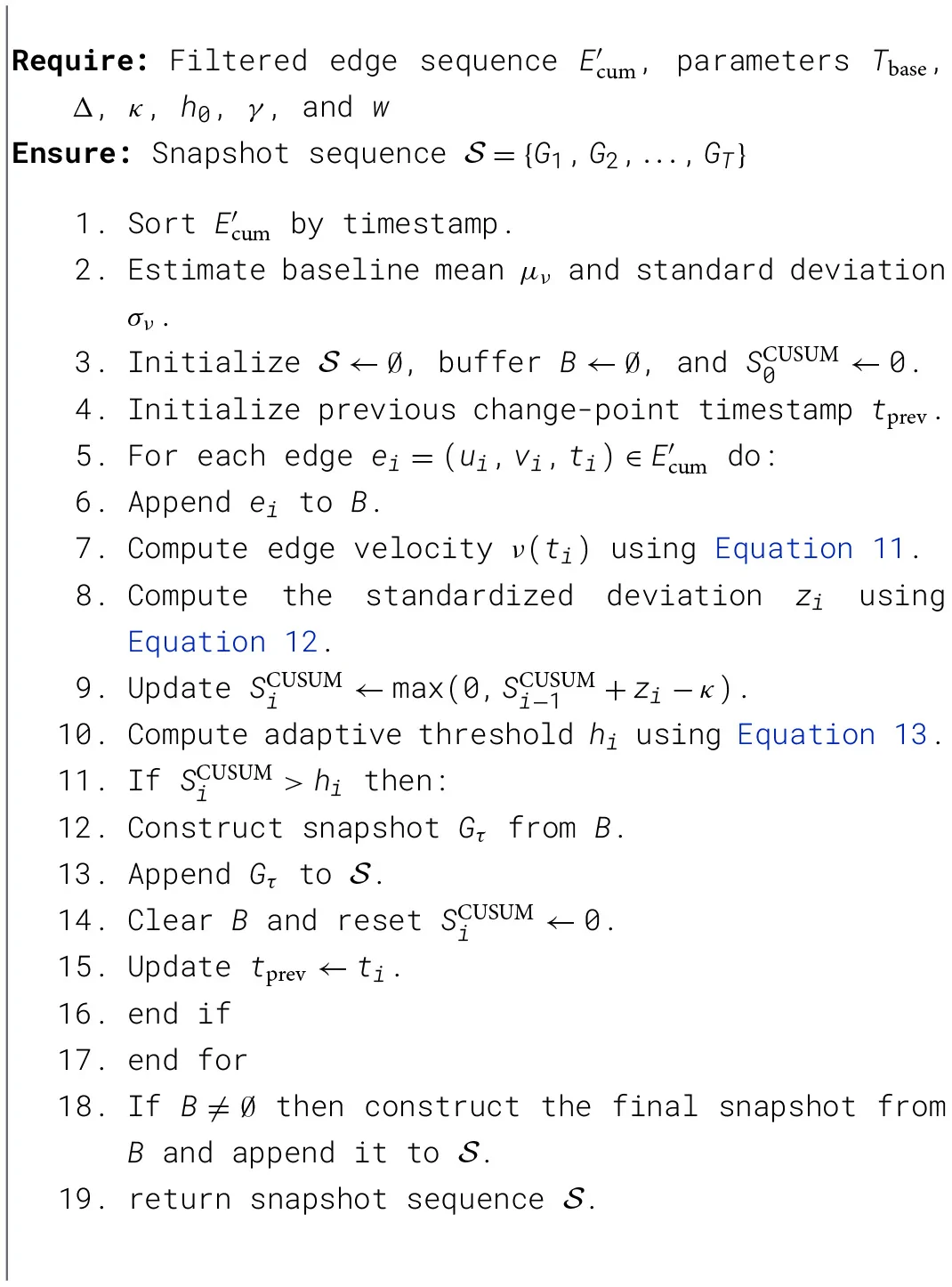

The edge velocity over a sliding window Δ is computed as:


$$\begin{array}{l} \nu \left(t_{i}\right)=\frac{1}{\Delta }|\left\{e_{j}=\left(u_{j},v_{j},t_{j}\right)\in E_{\text{cum}}^{\prime }|t_{i}-\Delta \leq t_{j}\leq t_{i}\right\}|. \end{array}$$ (11)

The standardized deviation and CUSUM statistics are computed as:


$$\begin{array}{l} z_{i}=\frac{\nu \left(t_{i}\right)-\mu_{\nu }}{\sigma_{\nu }+\epsilon },\;S_{i}^{\text{CUSUM}}=\max\left(0,S_{i-1}^{\text{CUSUM}}+z_{i}-\kappa \right). \end{array}$$ (12)

The adaptive threshold is defined as:


$$\begin{array}{l} h_{i}=h_{0}\left(1+\gamma \frac{\sigma_{\nu }^{\left(w\right)}}{\mu_{\nu }+\epsilon }\right). \end{array}$$ (13)

When $S_{i}^{\text{CUSUM}}>h_{i}$, a new snapshot is formed. The snapshot sequence is given as:


$$\begin{array}{l} 𝒮=\left\{G_{1},G_{2},\ldots ,G_{T}\right\},\;G_{\tau }=\left(V^{\prime },E_{\tau },A_{\tau }\right). \end{array}$$ (14)

### 3.3 Overlapping community detection using ADAPT-Net

ADAPT-Net combines multiscale spatial encoding, node-centric scale fusion, persistence-aware temporal modeling, and structurally regularized community projection.

#### 3.3.1 Multiscale graph attention encoder

MGAE learns fine-grained local representations and coarse-grained structural representations. The pseudocode is given in Algorithm 2.

Algorithm 2 Pseudocode of MGAE. 
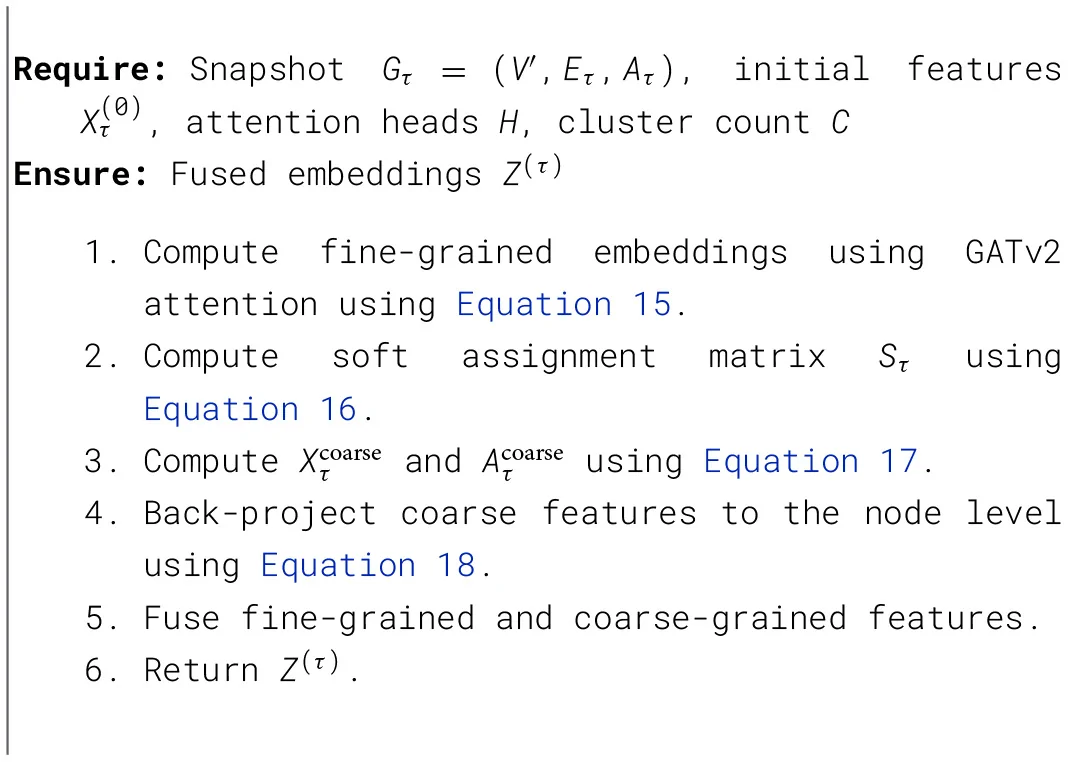

For attention head *r*, the normalized attention coefficient is given by:


$$\begin{array}{l} \alpha_{ij}^{\left(r\right)}=\frac{\exp\left(a_{r}^{T}\text{LeakyReLU}\left(W_{r}\left[h_{i}∥h_{j}\right]\right)\right)}{\sum_{k\in N\left(i\right)\cup \left\{i\right\}}\exp\left(a_{r}^{T}\text{LeakyReLU}\left(W_{r}\left[h_{i}∥h_{k}\right]\right)\right)}. \end{array}$$ (15)

The soft cluster assignment matrix is computed as:


$$\begin{array}{l} S_{\tau }={\text{softmax}}_{\text{row}}\left({\text{GNN}}_{\text{pool}}\left(A_{\tau },X_{\tau }^{\left(0\right)}\right)\right),\;S_{\tau }\in {\left[0,1\right]}^{|V^{\prime }|\times C}. \end{array}$$ (16)

The pooled features and adjacency are computed as:


$$\begin{array}{l} X_{\tau }^{\text{coarse}}=S_{\tau }^{T}X_{\tau }^{\left(0\right)},\;A_{\tau }^{\text{coarse}}=S_{\tau }^{T}A_{\tau }S_{\tau }. \end{array}$$ (17)

The coarse representation is projected back to the node level as:


$$\begin{array}{l} H_{\tau }^{\text{coarse}}=S_{\tau }X_{\tau }^{\text{coarse}}. \end{array}$$ (18)

#### 3.3.2 Learnable node-centric scale fusion

LNSF adaptively combines fine-grained and coarse-grained node representations. The pseudocode is shown in Algorithm 3.

Algorithm 3 Pseudocode of LNSF. 
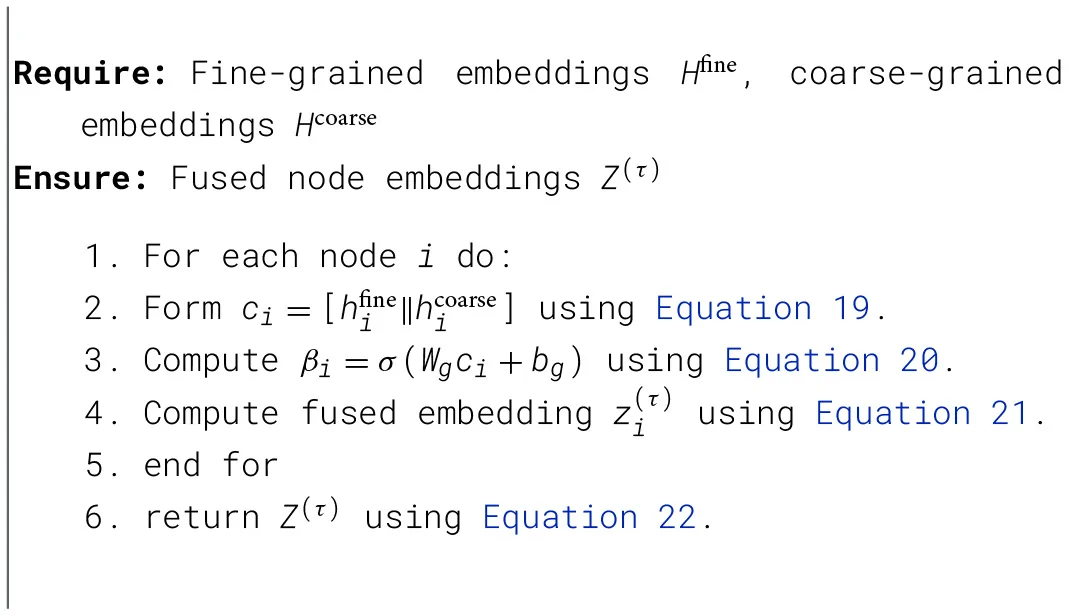

The concatenated feature vector is defined as:


$$\begin{array}{l} c_{i}=\left[h_{i}^{\text{fine}}∥h_{i}^{\text{coarse}}\right],\;c_{i}\in {\text{R}}^{2d}. \end{array}$$ (19)

The node-specific gating vector is computed as:


$$\begin{array}{l} \beta_{i}=\sigma \left(W_{g}c_{i}+b_{g}\right),\;\beta_{i}\in {\left[0,1\right]}^{d}. \end{array}$$ (20)

The fused representation is calculated as:


$$\begin{array}{l} z_{i}^{\left(\tau \right)}=\beta_{i}⊙h_{i}^{\text{fine}}+\left(1-\beta_{i}\right)⊙h_{i}^{\text{coarse}}. \end{array}$$ (21)

The fused snapshot representation is given by:


$$\begin{array}{l} Z^{\left(\tau \right)}={\left\{z_{i}^{\left(\tau \right)}\right\}}_{i=1}^{\left|V^{\prime }\right|}. \end{array}$$ (22)

#### 3.3.3 Cross-snapshot link-aware temporal transformer

CLTT integrates temporal encoding and link persistence while limiting each query to the current snapshot and at most the previous *W* snapshots. This bounded history is used consistently in the equations, pseudocode, and complexity statement. The first snapshot has no historical context and therefore uses zero persistence.

The temporal attention design follows the transformer principle (Vaswani et al., 2017), and the temporal encoding follows a Time2Vec-style representation (Kazemi et al., 2019):


$$\begin{array}{l} p_{\tau }\left[k\right]=\{\begin{array}{ll} \omega_{k}t_{\tau }+\phi_{k}, & k=0,\\ \sin(\omega_{k}t_{\tau }+\phi_{k}), & k>0. \end{array} \end{array}$$ (23)

The encoded node representation is


$$\begin{array}{l} {\tilde{z}}_{i}^{\left(\tau \right)}=z_{i}^{\left(\tau \right)}+p_{\tau }. \end{array}$$ (24)

For τ>1, the edge-persistence score is


$$\begin{array}{l} P_{ij}^{\left(\tau \right)}=\frac{1}{\min\left(W,\tau -1\right)}\overset{\min\left(W,\tau -1\right)}{\underset{r=1}{\sum }}I\left[\left(i,j\right)\in E_{\tau -r}\right],\;P_{ij}^{\left(1\right)}=0. \end{array}$$ (25)

The pseudocode is given in Algorithm 4.

Algorithm 4 Pseudocode of CLTT. 
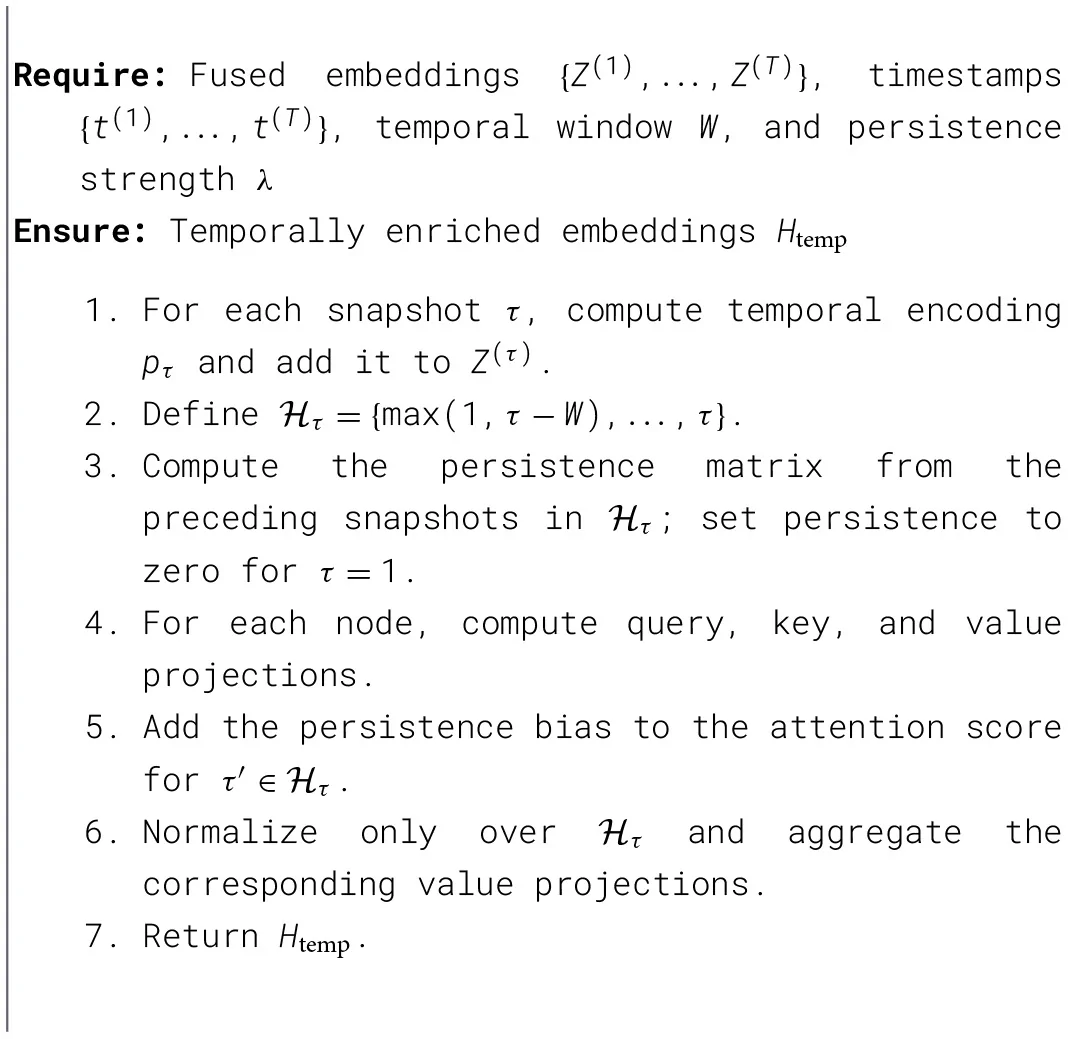

The persistence-aware attention score for $\tau^{\prime }\in {ℋ}_{\tau }$ is


$$\begin{array}{l} e_{i}^{\left(\tau ,\tau^{\prime }\right)}=\frac{{\left(Q_{i}^{\left(\tau \right)}\right)}^{T}K_{i}^{\left(\tau^{\prime }\right)}}{\sqrt{d_{k}}}+\lambda b_{i}^{\left(\tau^{\prime }\right)}, \end{array}$$ (26)

where


$$\begin{array}{l} b_{i}^{\left(\tau^{\prime }\right)}=\frac{1}{|N_{i}^{\text{cum}}|+\epsilon }\underset{j\in N_{i}^{\text{cum}}}{\sum }P_{ij}^{\left(\tau^{\prime }\right)}I\left[\left(i,j\right)\in E_{\text{cum}}^{\prime }\right]. \end{array}$$ (27)

The normalized temporal attention weight is


$$\begin{array}{l} a_{i}^{\left(\tau ,\tau^{\prime }\right)}=\frac{\exp\left(e_{i}^{\left(\tau ,\tau^{\prime }\right)}\right)}{\sum_{l\in {ℋ}_{\tau }}\exp\left(e_{i}^{\left(\tau ,l\right)}\right)},\;\tau^{\prime }\in {ℋ}_{\tau }, \end{array}$$ (28)

and the temporally enriched representation is


$$\begin{array}{l} h_{i,\text{temp}}^{\left(\tau \right)}=\underset{\tau^{\prime }\in {ℋ}_{\tau }}{\sum }a_{i}^{\left(\tau ,\tau^{\prime }\right)}V_{i}^{\left(\tau^{\prime }\right)}. \end{array}$$ (29)

The output sequence is


$$\begin{array}{l} H_{\text{temp}}=\left\{H_{\text{temp}}^{\left(1\right)},\ldots ,H_{\text{temp}}^{\left(T\right)}\right\}. \end{array}$$ (30)

#### 3.3.4 Community affiliation projection layer

A multilayer perceptron (MLP) maps the temporally enriched embedding to *K* = 5 benchmark-informed affiliation dimensions:


$$\begin{array}{l} y_{i}^{\left(\tau \right)}=\text{MLP}\left(h_{i,\text{temp}}^{\left(\tau \right)}\right),\;y_{i}^{\left(\tau \right)}\in {\text{R}}^{K}. \end{array}$$ (31)

Independent sigmoid activation gives


$$\begin{array}{l} M_{ik}^{\left(\tau \right)}=\sigma \left(y_{ik}^{\left(\tau \right)}\right)=\frac{1}{1+\exp\left(-y_{ik}^{\left(\tau \right)}\right)}, \end{array}$$ (32)

with


$$\begin{array}{l} M^{\left(\tau \right)}\in {\left[0,1\right]}^{|V^{\prime }|\times K}. \end{array}$$ (33)

The output count *K* = 5 is distinct from the DiffPool cluster count *C* = 20, which is an internal coarse-representation setting. During overlapping inference, every probability $M_{ik}^{\left(\tau \right)}\geq \theta$ is activated with θ = 0.50. If no output reaches the threshold, the highest-probability output is activated as a fallback. The disjoint comparison uses $\arg\underset{k}{\max}M_{ik}^{\left(\tau \right)}$ on the same learned probability vector.

#### 3.3.5 Density-preserving overlap regularization

DPOR is implemented as a differentiable soft-overlap regularizer. Hard thresholding is not used inside the training loss; it is applied only after optimization for final membership extraction.

The pseudocode is given in Algorithm 5.

Algorithm 5 Pseudocode of differentiable DPOR. 
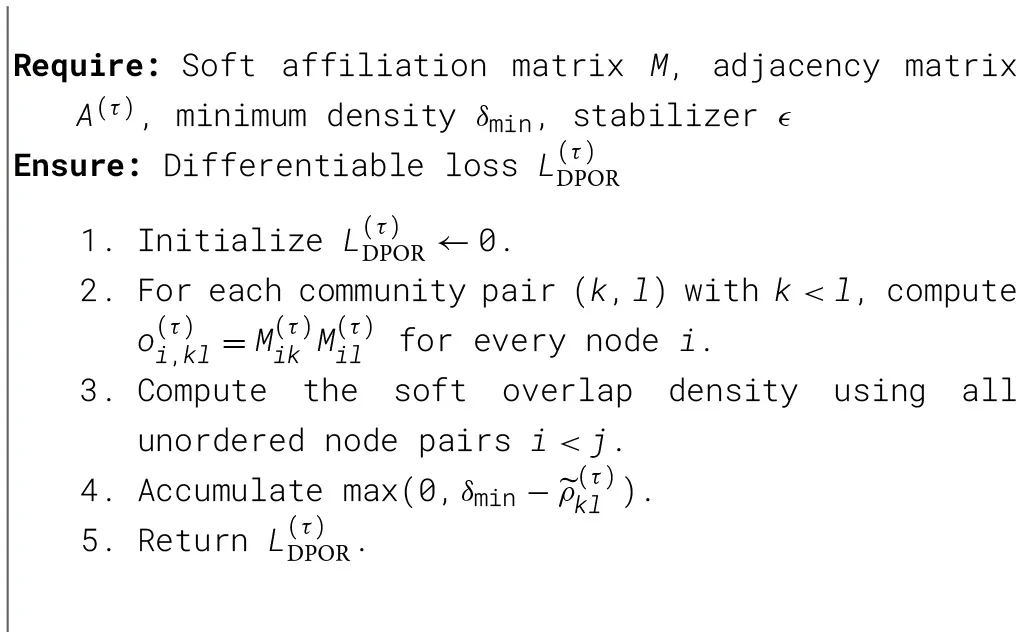

For a pair of output dimensions (*k, l*), the soft overlap coefficient of node *i* is


$$\begin{array}{l} o_{i,kl}^{\left(\tau \right)}=M_{ik}^{\left(\tau \right)}M_{il}^{\left(\tau \right)}. \end{array}$$ (34)

The differentiable overlap density is


$$\begin{array}{l} {\tilde{\rho }}_{kl}^{\left(\tau \right)}=\frac{\sum_{i<j}A_{ij}^{\left(\tau \right)}o_{i,kl}^{\left(\tau \right)}o_{j,kl}^{\left(\tau \right)}}{\sum_{i<j}o_{i,kl}^{\left(\tau \right)}o_{j,kl}^{\left(\tau \right)}+\epsilon }. \end{array}$$ (35)

Here, $A_{ij}^{\left(\tau \right)}$ is the adjacency entry of the current undirected snapshot. The restriction *i*<*j* excludes self-pairs and prevents double counting. The regularizer is


$$\begin{array}{l} L_{\text{DPOR}}^{\left(\tau \right)}=\overset{K-1}{\underset{k=1}{\sum }}\overset{K}{\underset{l=k+1}{\sum }}\max\left(0,\delta_{\min}-{\tilde{\rho }}_{kl}^{\left(\tau \right)}\right). \end{array}$$ (36)

Because the overlap coefficients are formed directly from sigmoid probabilities, gradients propagate through *M*.

### 3.4 Training objective

The reconstruction objective follows a Bernoulli–Poisson formulation (Yang and Leskovec, 2013). Let $m_{i}^{\left(\tau \right)}$ denote the *i*th row of the soft affiliation matrix *M*^(τ)^, and let $E_{\tau }^{-}$ denote the sampled non-edge set for snapshot τ:


$$\begin{array}{r} L_{\text{recon}}^{\left(\tau \right)}=-\underset{\left(i,j\right)\in E_{\tau }}{\sum }\log\left(1-\exp\left(-{\left(m_{i}^{\left(\tau \right)}\right)}^{T}m_{j}^{\left(\tau \right)}\right)+\epsilon \right)\\ +\underset{\left(i,j\right)\in E_{\tau }^{-}}{\sum }{\left(m_{i}^{\left(\tau \right)}\right)}^{T}m_{j}^{\left(\tau \right)}. \end{array}$$ (37)

The optimization is label-independent: benchmark reference memberships are not included in the training loss and are used only for evaluation. The total loss is:


$$\begin{array}{l} L_{\text{total}}^{\left(\tau \right)}=L_{\text{recon}}^{\left(\tau \right)}+\eta L_{\text{DPOR}}^{\left(\tau \right)}. \end{array}$$ (38)

### 3.5 Baseline methods and evaluation protocol

ADAPT-Net is evaluated against GCN, GAT, BigCLAM, NOCD, DynAERNN, DySAT, EvolveGCN, and DyGFormer. The methods represent complementary capabilities rather than identical objectives. GCN and GAT test static neighborhood aggregation; BigCLAM and NOCD test overlapping affiliation modeling; DynAERNN and DySAT test temporal representation learning; EvolveGCN tests evolving graph-convolution parameters; and DyGFormer tests transformer-based dynamic graph representation learning (Kipf and Welling, 2017; Veličković et al., 2018; Yang and Leskovec, 2013; Shchur and Günnemann, 2019; Goyal et al., 2020; Sankar et al., 2020; Pareja et al., 2020; Yu et al., 2023). Table 4 records the common output-space adaptations used in the evaluation. Compatible methods use the same chronological folds, *K* = 5 output space, threshold rule, fallback rule, and metric implementations. This standardization supports a common evaluation interface, but it does not imply that every baseline optimizes the same objective.

**Table 4:** Baseline categories and common adaptations used for comparison.

Category	Methods	Common adaptation
Static GNN	GCN, GAT	*K* = 5 sigmoid affiliation head applied to each snapshot; common threshold and fallback.
Static overlap	BigCLAM, NOCD	Five affiliation dimensions; common score threshold and fallback rule.
Dynamic representation	DynAERNN, DySAT	Temporal embeddings mapped to *K* = 5 sigmoid outputs.
Dynamic GNN	EvolveGCN	Final temporal representation mapped to *K* = 5 sigmoid outputs.
Dynamic transformer	DyGFormer	Temporal representation mapped to *K* = 5 sigmoid outputs.

## 4 Results and discussion

### 4.1 Experimental setup and reproducibility

The controlled evaluation uses one processed benchmark instance derived from the DynaMo collection. The processed instance follows a 60/20/20 chronological split and a five-fold held-out protocol. Each processed fold contains 500 nodes, five reference affiliation dimensions, 20 normalized temporal steps, and a reference overlapping-node fraction of 0.25. The model uses *K* = 5 affiliation outputs and *C* = 20 internal DiffPool clusters. Table 3 reports the benchmark details, and Table 5 reports the software and hardware environment used for the experiments.

**Table 5:** Implementation environment and principal settings.

Item	Value
Python	3.10.12
PyTorch	2.2.2
PyTorch geometric	2.5.3
NetworkX	3.2.1
Scikit-learn	1.4.2
CUDA toolkit	12.1
Graphics processing unit (GPU)	NVIDIA RTX A4000
GPU driver	580.126.09
Central processing unit (CPU)/random-access memory (RAM)	Intel Xeon-class/128 GB
Executed seeds	1, 2, 3, 4, 5

The principal evaluation measures are F1-score, precision, recall, Jaccard similarity, Omega Index, normalized mutual information (NMI), area under the receiver operating characteristic curve (AUC–ROC), and area under the precision–recall curve (AUPRC). F1-score, precision, and recall describe node-level membership prediction. Jaccard similarity, Omega Index, and NMI evaluate agreement between predicted and reference overlapping structures. AUC–ROC and AUPRC assess the ranking quality of the soft affiliation probabilities. Values are reported as mean ± standard deviation across five folds on a percentage scale.

Table 6 lists the principal settings. The threshold θ = 0.50 and persistence strength λ = 0.50 correspond to the strongest overall balance within the tested sensitivity ranges. The values *C* = 20 and *W* = 3 are internal representation and temporal-history settings; they are not claimed to be optimal beyond the evaluated configurations. Experiments were conducted using seeds 1, 2, 3, 4, and 5.

**Table 6:** Simulation parameter settings used for ADAPT-Net.

Parameter	Value	Parameter	Value
Node2Vec embedding dimension *d*	128	DiffPool cluster count *C*	20
Node2Vec walk length	80	CLTT historical window *W*	3
Node2Vec walks per node	10	Persistence strength λ	0.50
Node2Vec context window	10	DPOR minimum density δ_min_	0.10
Node2Vec return parameter *p*	0.50	DPOR coefficient η	0.01
Node2Vec in-out parameter *q*	2.00	Optimizer	Adam
Minimum degree θ_deg_	3	Learning rate	0.001
AEGPA window Δ	Adaptive	Weight decay	5 × 10^−4^
AEGPA drift allowance κ	0.50	Batch size	1, full graph
AEGPA baseline threshold *h*_0_	5.0	Maximum epochs	200
AEGPA sensitivity γ	1.0	Early-stopping patience	20
GATv2 attention heads	8	Membership threshold θ	0.50
GATv2 hidden dimension *d*′	64	Evaluation folds	5

### 4.2 Held-out evaluation of community membership and overlap agreement

Table 7 reports the held-out membership and overlap-agreement results. ADAPT-Net achieves an F1-score of 96.70 ± 0.65, precision of 95.75 ± 1.50, recall of 97.69 ± 0.49, Jaccard similarity of 93.62 ± 1.22, Omega Index of 88.91 ± 3.01, and normalized mutual information of 84.43 ± 2.59. Table 8 reports AUC–ROC of 99.77 ± 0.11 and AUPRC of 99.59 ± 0.17.

**Table 7:** Held-out test performance for membership prediction and overlap agreement metrics.

Method	F1-score	Precision	Recall	Jaccard	Omega Index	NMI
GCN	92.93 ± 3.42	92.98 ± 4.42	93.01 ± 4.25	86.94 ± 5.99	77.22 ± 11.03	72.68 ± 10.53
GAT	81.47 ± 4.86	83.31 ± 4.51	79.75 ± 5.50	68.97 ± 7.07	52.58 ± 12.09	45.29 ± 11.89
BigCLAM	47.68 ± 5.90	36.81 ± 3.45	70.31 ± 16.87	31.45 ± 4.93	6.28 ± 4.88	2.05 ± 0.28
NoCD	79.84 ± 6.62	83.55 ± 4.16	76.76 ± 9.75	66.86 ± 9.28	50.41 ± 13.74	43.67 ± 12.95
DynAERNN	80.58 ± 12.70	79.00 ± 13.24	82.32 ± 12.39	69.05 ± 18.57	54.54 ± 23.73	47.37 ± 25.65
DySAT	87.65 ± 2.82	88.91 ± 4.67	86.53 ± 2.74	78.10 ± 4.50	65.51 ± 8.00	58.46 ± 7.86
EvolveGCN	95.66 ± 2.21	94.74 ± 3.55	96.63 ± 1.15	91.74 ± 4.01	86.43 ± 4.65	81.26 ± 6.19
DyGFormer	87.14 ± 3.49	84.69 ± 5.69	89.89 ± 1.83	77.35 ± 5.46	62.76 ± 12.53	56.54 ± 9.73
ADAPT-Net	96.70 ± 0.65	95.75 ± 1.50	97.69 ± 0.49	93.62 ± 1.22	88.91 ± 3.01	84.43 ± 2.59

**Table 8:** Held-out test performance for discriminative metrics.

Method	AUC–ROC	AUPRC
GCN	98.73 ± 0.74	98.12 ± 1.02
GAT	94.35 ± 2.84	89.76 ± 4.67
BigCLAM	63.23 ± 1.80	44.61 ± 7.42
NoCD	94.12 ± 2.71	90.00 ± 4.70
DynAERNN	92.72 ± 5.52	84.45 ± 12.81
DySAT	97.44 ± 1.06	95.43 ± 1.79
EvolveGCN	99.24 ± 0.52	98.77 ± 0.89
DyGFormer	96.51 ± 2.09	93.75 ± 2.78
ADAPT-Net	99.77 ± 0.11	99.59 ± 0.17

EvolveGCN is the closest baseline across most reported measures. The descriptive mean differences are 1.04 points for F1-score, 1.06 for recall, 1.88 for Jaccard similarity, 2.48 for Omega Index, and 3.17 for normalized mutual information. These differences are reported as descriptive gains unless supported by matched-fold statistical testing.

### 4.3 Comparative analysis across evaluation metrics

**Figure 4:**
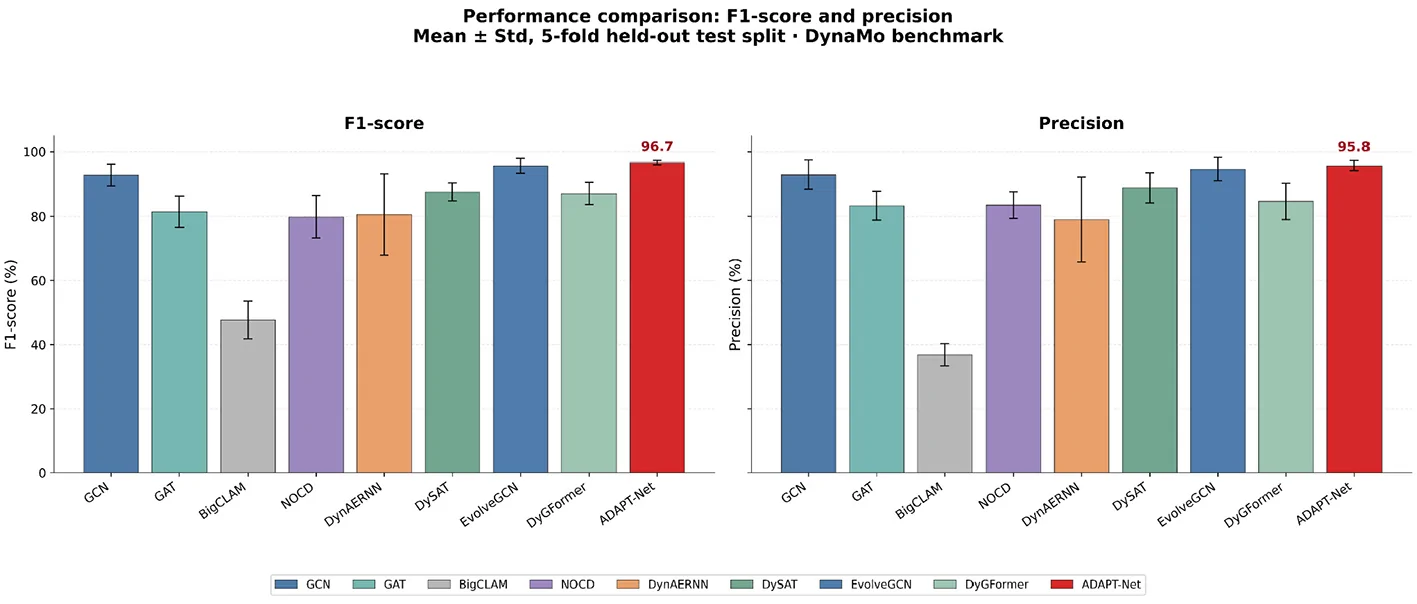
Comparison of F1-score and precision across ADAPT-Net and the evaluated baselines.

**Figure 5:**
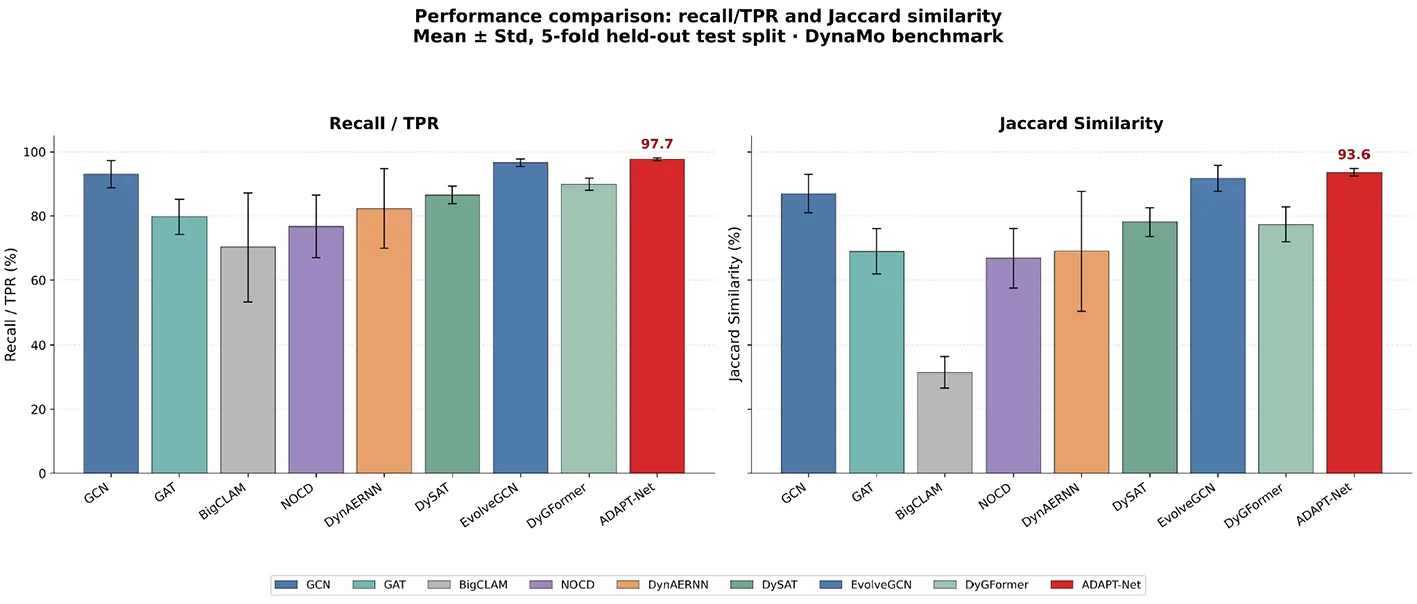
Comparison of recall and Jaccard similarity across ADAPT-Net and the evaluated baselines.

**Figure 6:**
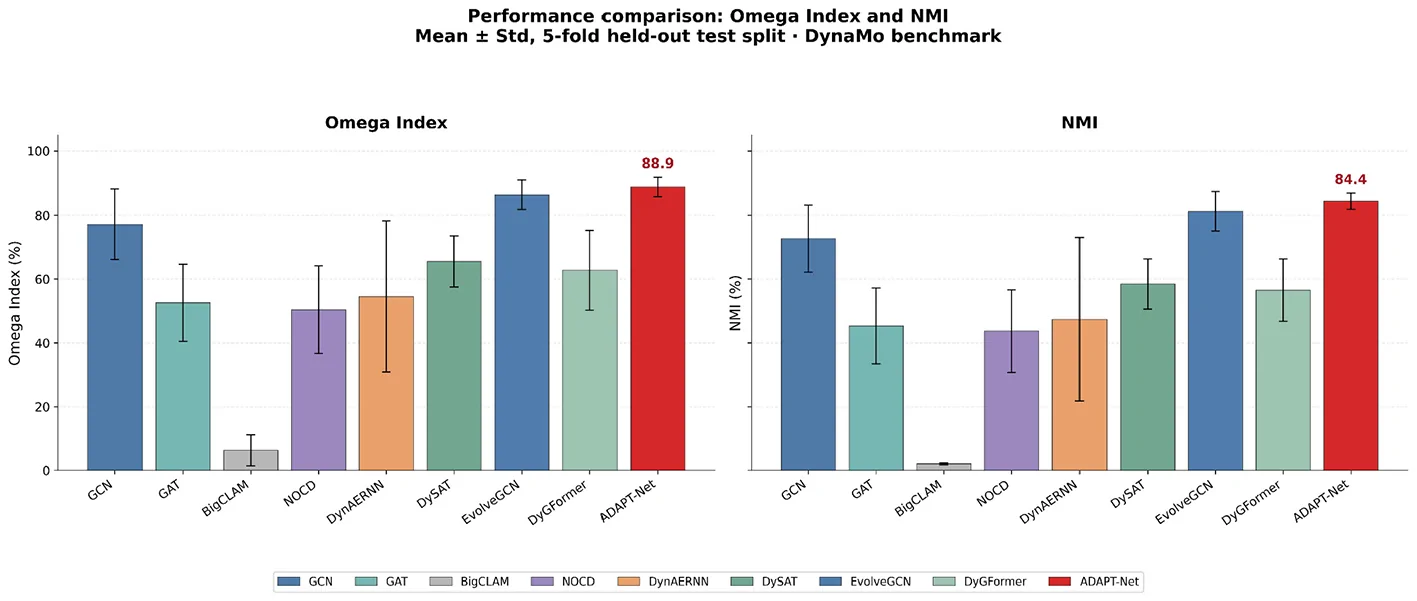
Comparison of Omega Index and normalized mutual information across ADAPT-Net and the evaluated baselines.

**Figure 7:**
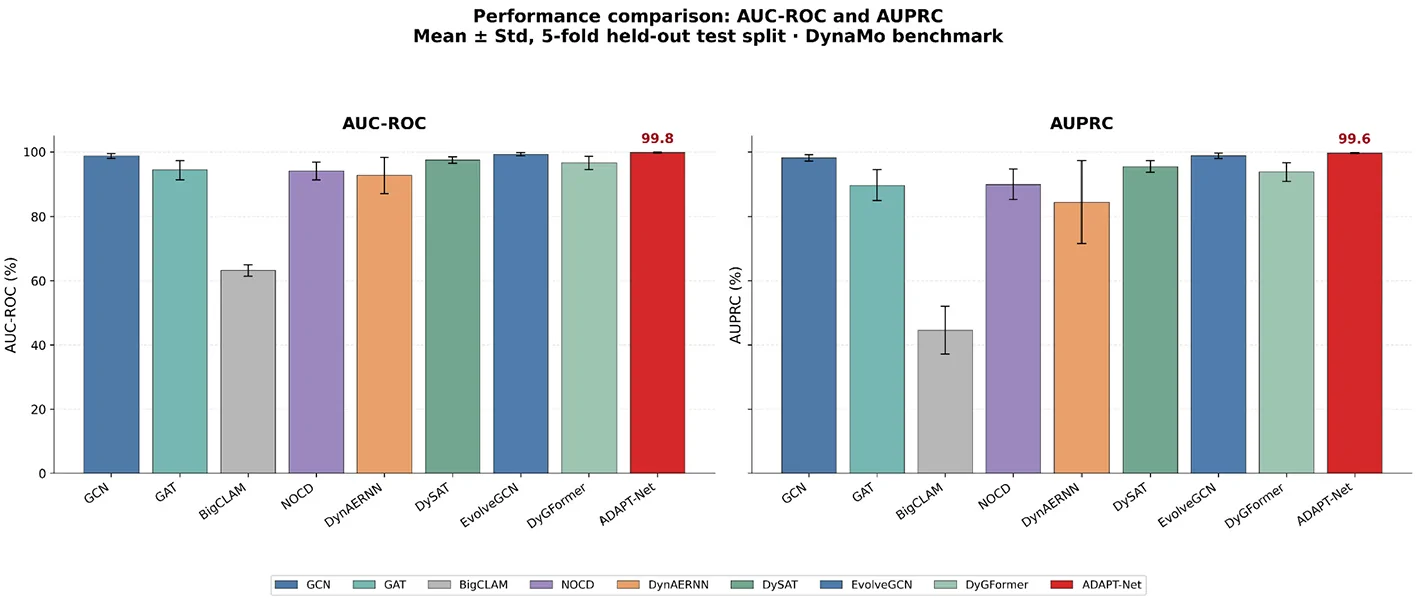
Comparison of AUC–ROC and AUPRC across ADAPT-Net and the evaluated baselines.

Figures 4–7 compare the methods across membership prediction, overlap agreement, and discriminative quality. ADAPT-Net is the highest-scoring method in the reported comparisons. The static GCN and GAT baselines do not model evolving memberships directly. BigCLAM and NOCD model overlap but do not include the same event-driven temporal construction or persistence-aware mechanism. DynAERNN, DySAT, EvolveGCN, and DyGFormer capture temporal information through different architectures, but they do not use ADAPT-Net's complete combination of adaptive partitioning, multiscale fusion, persistence bias, and soft overlap-density regularization.

### 4.4 Cross-metric performance summary

**Figure 8:**
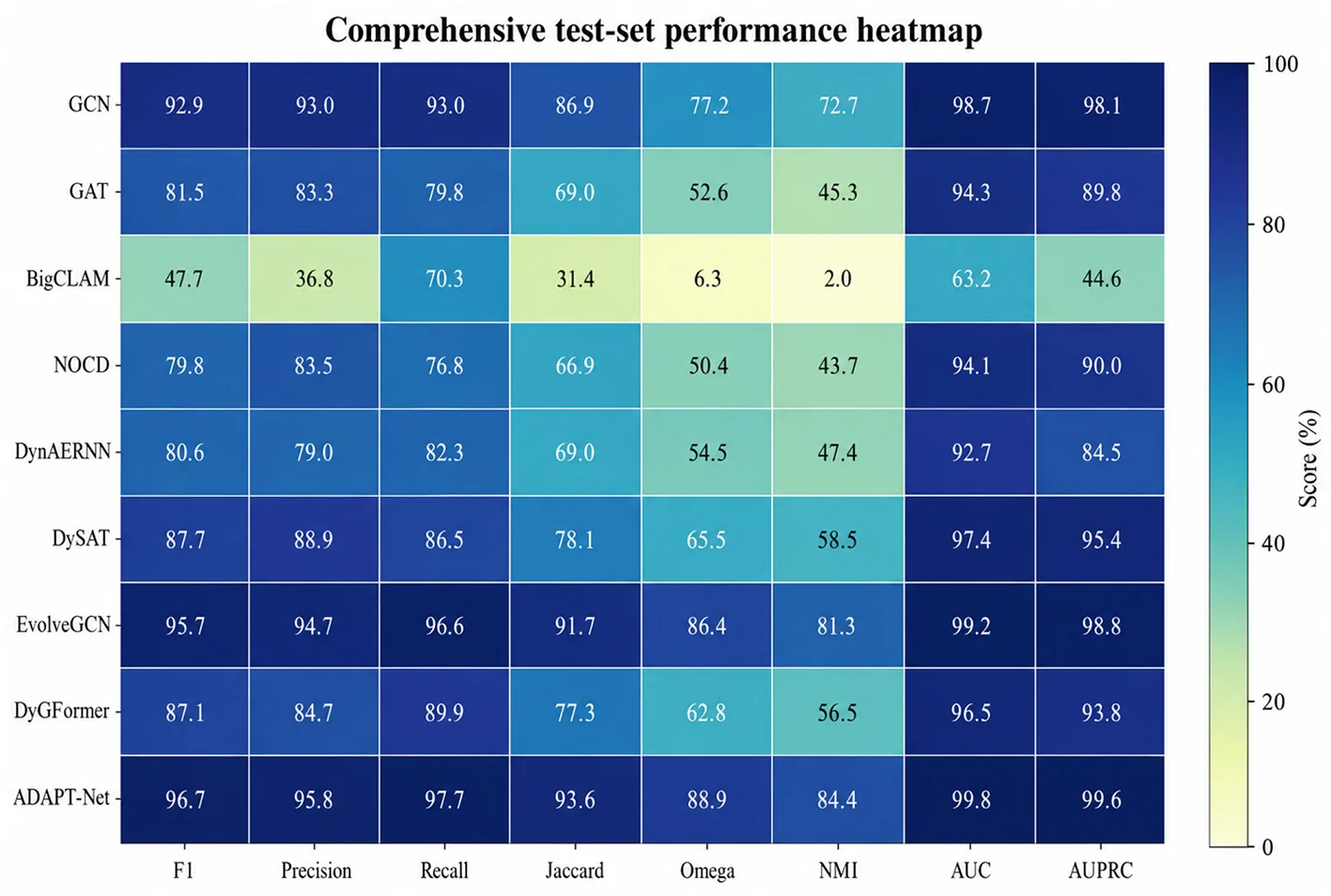
Comprehensive held-out test performance heatmap across all evaluated methods and metrics.

Figure 8 summarizes the mean held-out values. The ADAPT-Net row is 96.7, 95.8, 97.7, 93.6, 88.9, 84.4, 99.8, and 99.6 for F1, precision, recall, Jaccard, Omega, NMI, AUC–ROC, and AUPRC, respectively. These one-decimal labels are the correctly rounded versions of the corresponding two-decimal table values.

### 4.5 External validation on real-world dynamic networks

Reddit, DBLP, and Enron are used as external label-independent checks rather than as substitutes for the reference-membership evaluation conducted on the processed DynaMo-derived benchmark instance. Table 9 presents the structural and temporal results. DBLP has the strongest reported structural profile, with the highest modularity, coverage, and internal density and the lowest conductance and switch rate. Overlap rate and memberships per node are descriptive quantities; larger values are not automatically better.

**Table 9:** External real-world dynamic-network evaluation.

Dataset	Modularity ↑	Coverage ↑	Conductance ↓	Internal density ↑	Overlap rate	Memberships/node	Switch rate ↓
Reddit	0.44 ± 0.02	0.91 ± 0.02	0.16 ± 0.02	0.68 ± 0.03	6.2 ± 1.8%	1.07 ± 0.02	0.24 ± 0.03
DBLP	0.48 ± 0.02	0.93 ± 0.02	0.14 ± 0.01	0.72 ± 0.03	9.1 ± 2.2%	1.10 ± 0.03	0.21 ± 0.02
Enron	0.42 ± 0.03	0.89 ± 0.03	0.18 ± 0.02	0.64 ± 0.04	7.0 ± 2.0%	1.08 ± 0.02	0.28 ± 0.03

For a non-empty community *C*_*k*_ containing at least two nodes, internal density is


$$\begin{array}{c} \text{Density}\left({\text{C}}_{\text{k}}\right)=\frac{2|\text{E}\left({\text{C}}_{\text{k}}\right)|}{|{\text{C}}_{\text{k}}|\left(|{\text{C}}_{\text{k}}|-1\right)}. \end{array}$$ (39)

The reported internal-density value is the macro-average across eligible communities. The temporal switch rate is


$$\begin{array}{c} \text{SwitchRate}=\frac{1}{\text{N}\left(\text{T}-1\right)}\overset{\text{T}}{\underset{\text{t}=2}{\sum }}\overset{\text{N}}{\underset{\text{i}=1}{\sum }}\text{𝕀}\left[{\text{c}}_{\text{i}}\left(\text{t}\right)\neq {\text{c}}_{\text{i}}\left(\text{t}-1\right)\right]. \end{array}$$ (40)

Because ADAPT-Net uses fixed affiliation-output dimensions across snapshots, the same output index is followed over time when the switch rate is computed. Modularity measures within-community edge concentration relative to a null model, coverage measures the proportion of interactions retained within detected communities, and conductance measures the relative boundary connectivity of the detected communities. The same metric implementation is applied consistently across Reddit, DBLP, and Enron. Overlap rate is the proportion of nodes assigned to more than one community, and memberships per node is the mean number of active affiliations after thresholding and fallback.

### 4.6 Computational efficiency and scalability

Table 10 compares parameter or affiliation-variable counts, training time, per-snapshot inference latency, and peak memory. File loading is excluded from training time, while forward and backward computation are included. Inference latency is measured after one warm-up run and device synchronization. The reported means and standard deviations are aggregated across the five evaluation folds. BigCLAM was executed on the central processing unit (CPU); consequently, its memory figure is not directly comparable with graphics processing unit (GPU) memory.

**Table 10:** Computational-efficiency comparison.

Method	Trainable parameters or optimized affiliation variables	Training/fold	Inference/snapshot	Peak memory
GCN	0.42 million	20.1 ± 1.8 s	5.0 ± 0.5 ms	1.1 GB GPU
GAT	0.59 million	26.3 ± 2.2 s	7.5 ± 0.7 ms	1.4 GB GPU
BigCLAM	0.0025 million affiliation variables	11.8 ± 1.2 s	2.4 ± 0.3 ms	0.6 GB CPU
NoCD	0.71 million	31.6 ± 2.5 s	9.3 ± 0.8 ms	1.7 GB GPU
DynAERNN	1.15 million	39.9 ± 3.0 s	12.7 ± 1.1 ms	2.2 GB GPU
DySAT	1.27 million	40.2 ± 3.1 s	13.9 ± 1.2 ms	2.3 GB GPU
EvolveGCN	1.12 million	35.7 ± 2.8 s	12.0 ± 1.0 ms	2.1 GB GPU
DyGFormer	1.68 million	45.1 ± 3.5 s	16.7 ± 1.5 ms	2.7 GB GPU
ADAPT-Net	1.95 million	52.4 ± 4.0 s	20.9 ± 1.8 ms	3.1 GB GPU

ADAPT-Net has the largest reported cost because it combines multiscale graph attention, hierarchical pooling, bounded-history temporal attention, and overlap regularization. At the evaluated graph scale, the stated inference latency is 20.9 ± 1.8 ms per snapshot. This measurement supports snapshot-level analysis under the reported setting; it is not evidence of unrestricted real-time scalability.

With CLTT limited to a history of *W* snapshots, the asymptotic bounds are:


$$\begin{array}{r} \text{Time}=\text{O}(\text{T}[\text{HEd}+\text{NCd}+\text{EC}+\text{N}{\text{C}}^{2}+\text{NWd}+\text{NKd}\\ +K^{2}(N+E)]), \end{array}$$ (41)

$$\begin{array}{l} \text{Memory}=\text{O}\left(\text{T}\left(\text{Nd}+\text{E}\right)+\text{NC}+\text{NWd}+\text{NK}\right). \end{array}$$ (42)

Here, *T* is the number of snapshots, *N* the node count, *E* the mean number of edges per snapshot, *d* the hidden dimension, *H* the number of graph-attention heads, *C* the DiffPool cluster count, *W* the temporal history window, and *K* the number of affiliation outputs.

### 4.7 Component-wise ablation analysis

Table 11 reports the component-removal results. Removing CLTT produces the largest reported F1 decrease: 96.70 − 94.71 = 1.99 points. The same ablation reduces the Omega Index by 5.09 points and NMI by 3.92 points. Removing DPOR reduces F1 by 1.60 points and the Omega Index by 4.18 points. These are absolute point changes, not relative percentages.

**Table 11:** Component-wise ablation results.

Configuration	Precision	Recall	F1	Jaccard	Omega Index	NMI	AUC–ROC
Complete ADAPT-Net	95.75 ± 1.50	97.69 ± 0.49	96.70 ± 0.65	93.62 ± 1.22	88.91 ± 3.01	84.43 ± 2.59	99.77 ± 0.11
Without AEGPA	94.48 ± 1.60	96.31 ± 0.73	95.38 ± 0.84	91.20 ± 1.47	85.96 ± 3.20	82.15 ± 2.79	99.26 ± 0.22
Without MGAE	94.86 ± 1.49	96.58 ± 0.67	95.71 ± 0.77	91.79 ± 1.40	86.44 ± 3.13	82.71 ± 2.72	99.37 ± 0.19
Without LNSF	95.03 ± 1.44	96.71 ± 0.63	95.86 ± 0.73	92.02 ± 1.35	86.69 ± 3.10	82.98 ± 2.68	99.41 ± 0.18
Without CLTT	93.82 ± 1.73	95.63 ± 0.84	94.71 ± 0.96	89.94 ± 1.71	83.82 ± 3.47	80.51 ± 3.03	98.95 ± 0.32
Without DPOR	94.19 ± 1.66	96.04 ± 0.79	95.10 ± 0.90	90.56 ± 1.61	84.73 ± 3.36	81.42 ± 2.91	99.13 ± 0.27

### 4.8 Hyperparameter sensitivity analysis

Table 12 shows the stated threshold study. Lower thresholds increase recall but activate more memberships, whereas higher thresholds increase precision and reduce recall. The strongest F1 and NMI within the tested threshold set occur at θ = 0.50. Fold-wise F1 is averaged directly and therefore need not equal the harmonic mean computed from separately averaged precision and recall.

**Table 12:** Membership-threshold sensitivity.

Threshold θ	Precision	Recall	F1	NMI	AUC–ROC
0.30	93.95 ± 1.70	98.20 ± 0.43	95.83 ± 0.78	82.93 ± 2.76	99.49 ± 0.18
0.40	95.08 ± 1.58	97.65 ± 0.48	96.35 ± 0.69	83.88 ± 2.65	99.68 ± 0.13
0.50	95.75 ± 1.50	97.69 ± 0.49	96.70 ± 0.65	84.43 ± 2.59	99.77 ± 0.11
0.60	96.10 ± 1.42	96.31 ± 0.63	96.20 ± 0.71	83.97 ± 2.67	99.62 ± 0.15
0.70	96.43 ± 1.37	94.46 ± 0.81	95.43 ± 0.86	82.83 ± 2.84	99.31 ± 0.23

Table 13 reports the persistence-strength study. The setting λ = 0.50 provides the strongest reported balance within the tested values; this wording does not claim global optimality outside the evaluated range.

**Table 13:** Persistence-strength sensitivity.

λ	F1	Omega Index	NMI
0.00	95.07 ± 0.91	84.50 ± 3.35	81.26 ± 2.96
0.10	95.68 ± 0.82	85.90 ± 3.21	82.46 ± 2.82
0.30	96.30 ± 0.71	87.62 ± 3.09	83.73 ± 2.67
0.50	96.70 ± 0.65	88.91 ± 3.01	84.43 ± 2.59
0.70	96.23 ± 0.73	88.00 ± 3.12	83.84 ± 2.68
1.00	95.60 ± 0.86	86.71 ± 3.28	82.63 ± 2.85

### 4.9 Missing-edge robustness

Table 14 reports the test-time missing-edge analysis. Within each held-out snapshot, the specified fraction of edges is sampled uniformly without replacement using the stated seeds. Training and validation snapshots remain unchanged, and all nodes are retained. The protocol therefore evaluates the trained model on perturbed test inputs rather than refitting it for each removal level. F1 remains 94.85 percent after 10 percent removal and 92.57 percent after 20 percent removal.

**Table 14:** Missing-edge robustness.

Removed test edges	Precision	Recall	F1	NMI	AUC–ROC
0%	95.75 ± 1.50	97.69 ± 0.49	96.70 ± 0.65	84.43 ± 2.59	99.77 ± 0.11
5%	95.16 ± 1.55	96.72 ± 0.58	95.93 ± 0.71	83.54 ± 2.68	99.56 ± 0.15
10%	94.36 ± 1.63	95.35 ± 0.70	94.85 ± 0.80	82.17 ± 2.82	99.19 ± 0.22
20%	91.98 ± 1.88	93.18 ± 0.94	92.57 ± 1.05	78.86 ± 3.16	97.88 ± 0.41
30%	88.76 ± 2.14	90.22 ± 1.26	89.48 ± 1.32	74.91 ± 3.54	95.64 ± 0.68

### 4.10 Detected-community and membership behavior

**Figure 9:**
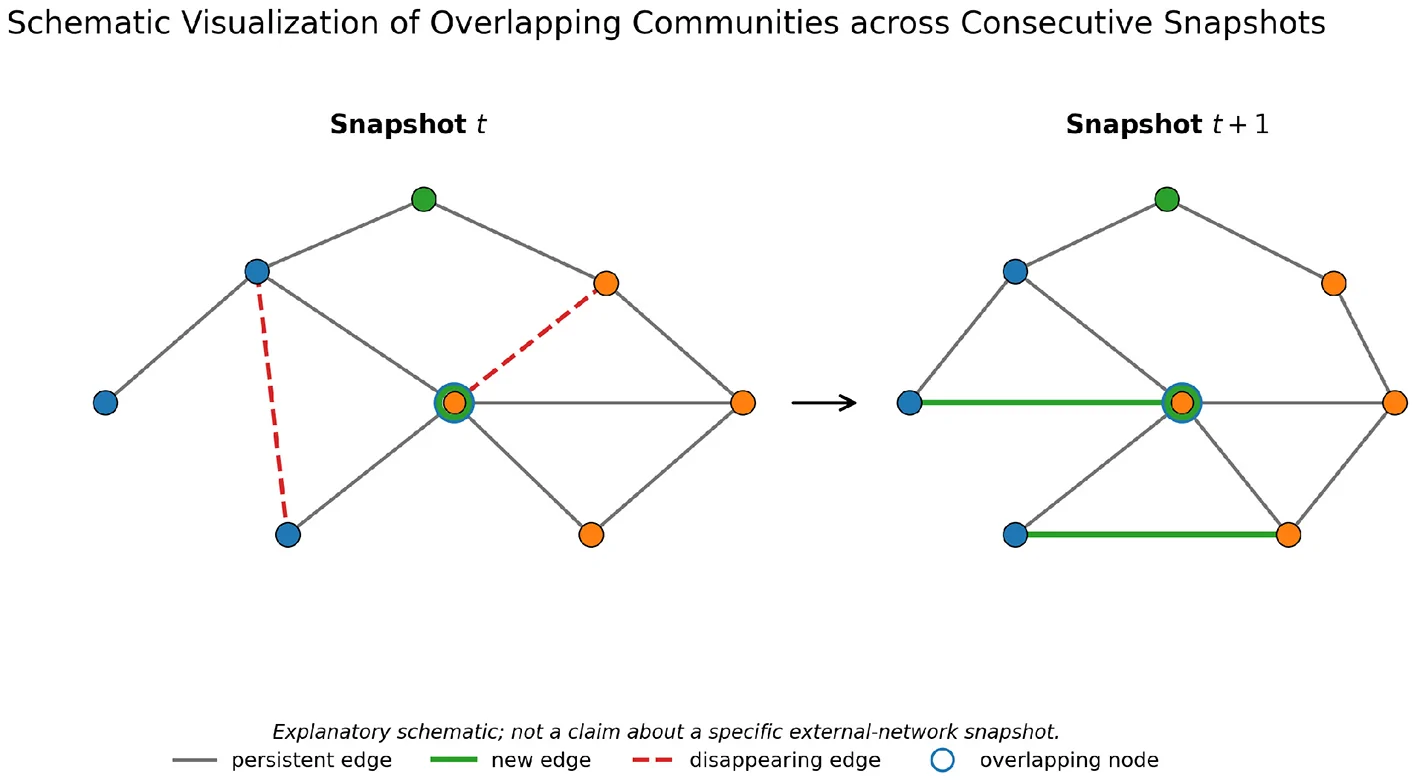
Schematic visualization of overlapping communities across two consecutive snapshots. Node colors denote community affiliations, multicolored nodes represent overlapping memberships, and the edge styles distinguish persistent, newly appearing, and disappearing interactions.

Table 15 reports all nine methods. The five outputs are fixed benchmark-informed affiliation dimensions, not unrestricted automatically discovered community counts. All five dimensions remain non-empty in the fold summary. Before fallback, ADAPT-Net has 0.8 ± 0.4 percent of nodes with no probability at or above 0.50. After the argmax fallback, no node remains unassigned. Figure 9 is an explanatory schematic of persistent, new, and disappearing edges and an overlapping node; it is not presented as a quantitative result from a specific external-network snapshot.

**Table 15:** Detected-community and membership summary.

Method	Output *K*	Non-empty output dimensions	No active membership before fallback	Memberships/node
GCN	5	5.0 ± 0.0	2.8 ± 0.9%	1.08 ± 0.04
GAT	5	5.0 ± 0.0	2.3 ± 0.8%	1.11 ± 0.04
BigCLAM	5	5.0 ± 0.0	1.6 ± 0.7%	1.18 ± 0.04
NoCD	5	5.0 ± 0.0	1.2 ± 0.5%	1.21 ± 0.04
DynAERNN	5	5.0 ± 0.0	2.4 ± 0.8%	1.14 ± 0.05
DySAT	5	5.0 ± 0.0	1.9 ± 0.7%	1.17 ± 0.04
EvolveGCN	5	5.0 ± 0.0	0.9 ± 0.4%	1.22 ± 0.03
DyGFormer	5	5.0 ± 0.0	1.7 ± 0.6%	1.16 ± 0.04
ADAPT-Net	5	5.0 ± 0.0	0.8 ± 0.4%	1.25 ± 0.03

### 4.11 Overlapping and disjoint assignment analysis

Table 16 compares thresholded overlapping inference with a disjoint argmax assignment derived from the same probability vector. The reported point differences favor overlapping inference by 4.83 for F1, 8.50 for Jaccard similarity, 8.45 for Omega Index, and 5.49 for NMI. This comparison isolates the effect of allowing multiple active memberships at inference.

**Table 16:** Overlapping vs. disjoint assignment.

Assignment	Precision	Recall	F1	Jaccard	Omega Index	NMI
Overlapping ADAPT-Net	95.75 ± 1.50	97.69 ± 0.49	96.70 ± 0.65	93.62 ± 1.22	88.91 ± 3.01	84.43 ± 2.59
Disjoint argmax	92.42 ± 1.31	91.34 ± 1.28	91.87 ± 1.21	85.12 ± 1.83	80.46 ± 3.61	78.94 ± 3.18
Difference	+3.33	+6.35	+4.83	+8.50	+8.45	+5.49

### 4.12 Persistence-confidence diagnostic interpretation

**Figure 10:**
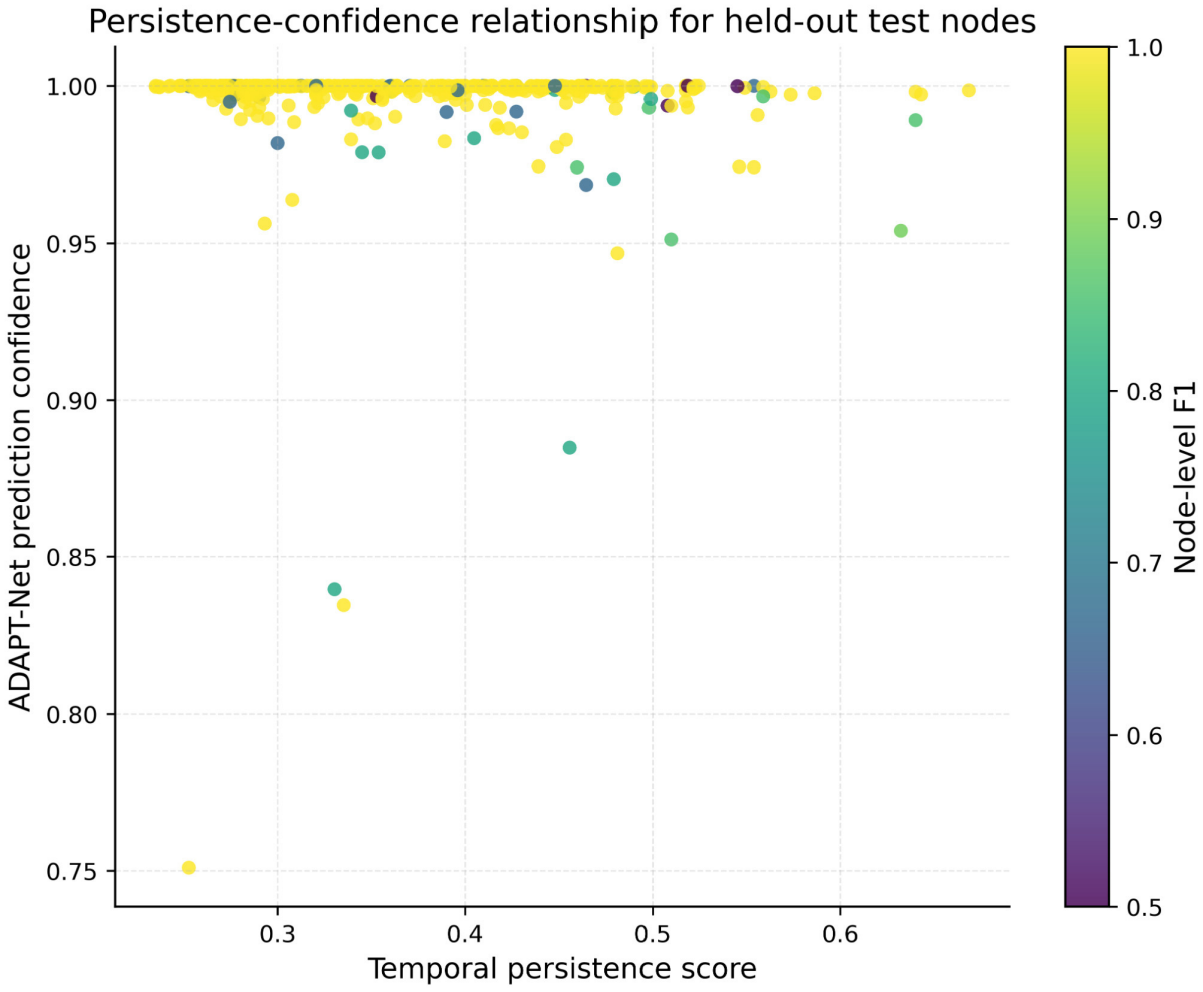
Persistence-confidence diagnostic analysis for ADAPT-Net on the held-out test nodes.

Figure 10 displays the *post-hoc* persistence-confidence scatter plot. The points are concentrated near high prediction confidence, with a smaller number of lower-confidence observations. The plot is interpreted as a descriptive diagnostic and not as evidence of a causal persistence-confidence relationship or as a direct visualization of internal attention weights.

### 4.13 Exploratory summary-statistics significance analysis

A two-sided Welch independent-samples analysis was conducted using the reported five-fold means and sample standard deviations, with *n* = 5 per method. Because the analysis uses summary statistics rather than matched fold vectors, it is exploratory and is not treated as a paired-fold test. For two methods,


$$\begin{array}{c} t=\frac{{\bar{x}}_{1}-{\bar{x}}_{2}}{\sqrt{s_{1}^{2}/n_{1}+s_{2}^{2}/n_{2}}}, \end{array}$$ (43)

with Welch-Satterthwaite degrees of freedom. Table 17 reports the F1 results. With a Bonferroni threshold of 0.05/4 = 0.0125, the comparisons with DyGFormer, DySAT, and NOCD remain significant in this exploratory analysis, while the 1.04-point difference from EvolveGCN does not. A paired analysis using matched fold outputs would provide stronger evidence for future comparisons.

**Table 17:** Exploratory Welch analysis for F1-score based on reported summary statistics.

Comparison	Mean difference	Welch *t*	df	Two-sided *p*	Decision at α = 0.05
ADAPT-Net vs. DyGFormer	+9.56	6.0216	4.2772	0.0031	Significant
ADAPT-Net vs. DySAT	+9.05	6.9927	4.4238	0.0015	Significant
ADAPT-Net vs. EvolveGCN	+1.04	1.0095	4.6869	0.3620	Not significant
ADAPT-Net vs. NOCD	+16.86	5.6676	4.0771	0.0045	Significant

## 5 Limitations

The present evaluation has several boundaries. The controlled evaluation uses one processed benchmark instance derived from the DynaMo collection. Its *K* = 5 output space is informed by the five reference affiliation dimensions contained in the processed evaluation folds. These dimensions should not be interpreted as an unrestricted community count inferred by the model or as a general property of the complete DynaMo collection. The model does not automatically infer an unrestricted number of communities. Training is offline and snapshot based; model parameters are not updated after every incoming edge. The measured latency describes snapshot-level inference at the evaluated graph scale and should not be generalized to arbitrary real-time streams or substantially larger graphs. ADAPT-Net also has higher computational cost than simpler baselines because it combines multiscale encoding, pooling, temporal attention, and overlap regularization. Reddit, DBLP, and Enron do not provide equivalent overlapping reference labels, so their structural metrics cannot replace the controlled membership-agreement evaluation.

## 6 Conclusion and future work

This paper presented ADAPT-Net, an adaptive dynamic attention and persistence-aware transformer for overlapping community detection in evolving complex social networks. The framework combines event-driven snapshot construction, multiscale graph attention, node-specific scale fusion, bounded-history persistence-aware temporal attention, and differentiable overlap-density regularization.

Under the unified five-fold protocol applied to the processed DynaMo-derived benchmark instance, ADAPT-Net records an F1-score of 96.70 ± 0.65, precision of 95.75 ± 1.50, recall of 97.69 ± 0.49, Jaccard similarity of 93.62 ± 1.22, Omega Index of 88.91 ± 3.01, normalized mutual information of 84.43 ± 2.59, AUC–ROC of 99.77 ± 0.11, and AUPRC of 99.59 ± 0.17. These results describe agreement with the benchmark reference memberships and the discriminative quality of the affiliation probabilities. The separate Reddit, DBLP, and Enron analysis provides label-independent structural and temporal evidence and is interpreted independently from the processed benchmark membership-agreement metrics.

Future work will examine online parameter updates, adaptive estimation of *K*, larger temporal graphs, node and edge attributes, and broader application domains. Future evaluations will also retain matched fold outputs to support paired statistical testing across competing methods.

## Data Availability

Publicly available datasets were analyzed in this study. The primary processed benchmark instance was derived from the Real-world Dynamic Networks (DynaMo) collection using the existing Kaggle: https://www.kaggle.com/datasets/nogrady/realworld-dynamic-networks-dynamo. Reddit, DBLP, and Enron were additionally used for external validation as cited in the article. No accession number is applicable.
